# Prion Protein Accumulation in Lipid Rafts of Mouse Aging Brain

**DOI:** 10.1371/journal.pone.0074244

**Published:** 2013-09-10

**Authors:** Federica Agostini, Carlos G. Dotti, Azucena Pérez-Cañamás, Maria Dolores Ledesma, Federico Benetti, Giuseppe Legname

**Affiliations:** 1 Laboratory of Prion Biology, Department of Neuroscience, Scuola Internazionale Superiore di Studi Avanzati (SISSA), Trieste, Italy; 2 Department of Molecular and Developmental Genetics, VIB Center for the Biology of Disease, K.U., Leuven, Leuven, Belgium; 3 Department of Human Genetics, K.U., Leuven, Leuven, Belgium; 4 Italian Institute of Technology, Trieste, Italy; 5 ELETTRA Laboratory, Sincrotrone Trieste S.C.p.A, AREA Science Park, Basovizza, Trieste, Italy; 6 Centro Biología Molecular Severo Ochoa (CSIC-UAM), Madrid, Spain; Ruhr University Bochum, Germany

## Abstract

The cellular form of the prion protein (PrP^C^) is a normal constituent of neuronal cell membranes. The protein misfolding causes rare neurodegenerative disorders known as transmissible spongiform encephalopathies or prion diseases. These maladies can be sporadic, genetic or infectious. Sporadic prion diseases are the most common form mainly affecting aging people. In this work, we investigate the biochemical environment in which sporadic prion diseases may develop, focusing our attention on the cell membrane of neurons in the aging brain. It is well established that with aging the ratio between the most abundant lipid components of rafts undergoes a major change: while cholesterol decreases, sphingomyelin content rises. Our results indicate that the aging process modifies the compartmentalization of PrP^C^. In old mice, this change favors PrP^C^ accumulation in detergent-resistant membranes, particularly in hippocampi. To confirm the relationship between lipid content changes and PrP^C^ translocation into detergent-resistant membranes (DRMs), we looked at PrP^C^ compartmentalization in hippocampi from acid sphingomyelinase (ASM) knockout (KO) mice and synaptosomes enriched in sphingomyelin. In the presence of high sphingomyelin content, we observed a significant increase of PrP^C^ in DRMS. This process is not due to higher levels of total protein and it could, in turn, favor the onset of sporadic prion diseases during aging as it increases the PrP intermolecular contacts into lipid rafts. We observed that lowering sphingomyelin in scrapie-infected cells by using fumonisin B1 led to a 50% decrease in protease-resistant PrP formation. This may suggest an involvement of PrP lipid environment in prion formation and consequently it may play a role in the onset or development of sporadic forms of prion diseases.

## Introduction

The cellular form of the prion protein (PrP), PrP^C^, is a glycosylphosphatidylinositol (GPI)-anchored protein present at the surface of cells, mainly expressed in the nervous system [1–4]. The protein was discovered due to its involvement in prion diseases. Prions, the causative agents of these maladies, appear in fact to be composed exclusively of a conformational isoform of PrP^C^ known as PrP^Sc^. The latter contains numerous β-sheet structures and tends to aggregate and form medium- to large-sized polymers [5–8].

Prion diseases are a group of rare neurodegenerative disorders that are progressive, fatal and at present incurable, leading to death within a few months to several years. Although the clinical profiles differ among distinct prion diseases, the characteristics of brain damage are similar and include extensive spongiform degeneration, widespread neuronal loss, synaptic alterations, atypical brain inflammation and the accumulation of protein aggregates [9]. A hallmark of prion diseases is their etiology: they can be sporadic, genetic and also infectious. The majority of cases are sporadic (around 85%) and the triggering factor is still unknown [10]. Sporadic prion diseases usually affect people between the ages of 45 and 75, and the average age of onset is around 65. The duration of the illness varies: for most people it lasts less than a year and may be as short as 6 weeks; in a minority of cases the illness can last up to 3 years.

Despite over twenty years of research, several important issues in the prion field remain unresolved. Most noticeably, both the physiological function of PrP^C^ and the molecular pathways leading to fatal neurodegeneration in prion diseases are poorly understood. Early studies on the cellular and disease-associated PrP have determined that both forms are tethered to cellular membranes via a GPI anchor [11]. Like many other GPI-anchored proteins, at steady state levels PrP^C^ has been shown to associate predominantly with lipid rafts [12]. The precise PrP^C^ to PrP^Sc^ conversion site is another aspect of prion biology that is still controversial. Some studies have reported that PrP^C^ appears to reach its surface localization and is subsequently internalized, leading to the conversion to PrP^Sc^ in intracellular compartments [13]. Others, instead, have suggested that the conversion of PrP^C^ to PrP^Sc^ takes place in lipid rafts. *In vitro* studies, using immortalized cell lines, have shown that lipid raft composition can influence prion conversion [14–17].

Additional investigations have reported that the ratio of major lipid components of lipid rafts changes during aging [18,19]. Cholesterol and sphingolipids play a key role in the organization of lipid rafts as well as in modulating their functions. Lipid rafts act as intracellular signaling platforms and, among other things, they are important for the differentiation and survival pathways in neurons [20,21]. Consequently, correct lipid homeostasis at the plasma membrane appears essential for cell survival and functioning. Changes in the cholesterol/sphingolipids ratio have been shown to accompany the brain aging process, influencing cellular pathways in a ligand-independent manner [21–25]. In agreement with these data from *in vitro* and *in vivo* animal studies, a moderate loss of brain cholesterol with age has also been described in humans [18].

Abnormalities in lipid rafts have also been found in several other diseases, including atherosclerosis, diabetes, cancer, muscular dystrophy and neurodegenerative disorders such as Alzheimer’s disease [26,27]. Furthermore, a deregulation of sphingolipid metabolism with consequent membrane disorganization has been described in several neurodegenerative disorders [28].

Altogether these observations highlight the importance of lipid homeostasis for the physiology of the plasma membrane and, consequently, of the whole cell. Therefore, we decided to investigate the effects of changes in lipid raft composition on PrP compartmentalization and prion formation. In particular, we focused our attention on physiological changes occurring in the aging brain, where sporadic prion diseases are more likely to arise in humans. We observed that variations in the cholesterol/sphingomyelin (SM) ratio, mimicking those occurring during aging, parallel the accumulation of PrP^C^ in detergent-resistant membranes (DRMs). These findings, together with additional results obtained from scrapie-infected cell lines, led us to hypothesize that the accumulation of PrP^C^ in DRMs during aging could be one of the factors triggering sporadic prion diseases in mammals.

## Materials and Methods

### Mice

#### Ethics Statement

All experiments were carried out in accordance with European regulations [European Community Council Directive, November 24, 1986 (86/609/EEC)]. Experimental procedures were notified to and approved by the Italian Ministry of Health, Directorate General for Animal Health (notification of 17 Sept. 2012). All experiments were approved by the local authority veterinary service and by SISSA Ethics Committee. Animals were killed by cervical dislocation.

Wild-type male C57BL/6J mice were obtained from the Charles River Laboratory.

A breeding colony of acid sphingomyelinase (ASM) knockout (KO) heterozygous C57BL/6J mice [29], kindly donated by Edward H. Schuchman (Mount Sinai School of Medicine, New York, NY), was established. The experiments were performed by comparing littermates of wild-type or ASMKO mice (5 months of age); the genotype was determined from genomic DNA in a PCR reaction.

### Membrane purification from tissue

Hippocampi from C57BL/6J male mice or ASMKO mice were dissected on ice, washed twice in cold PBS and homogenized in 25 mM MES Buffer, pH 7.0, 5 mM DTT, 2 mM EDTA and protease inhibitors (Roche), using a Dounce homogenizer and 10 passages through a 24-gauge syringe. Samples were centrifuged at 700 *g* for 10 min at 4°C and supernatants were considered as total extracts. A further centrifugation step was performed at 100,000 *g* for 1 hour at 4°C to pellet the membrane fraction (Optima Max Ultracentrifuge, Beckman Instruments). The resulting pellet was re-suspended in PBS + 0.2% SDS to be subsequently processed by Western blot. Protein concentration was determined using the Bio-Rad Protein Microassay.

### Detergent-resistant membrane purification from tissue

DRMs were prepared from total hippocampal extracts by Triton X-100 cold extraction; soluble and insoluble fractions were separated as described by Trovò et al. [23]. Briefly, total protein extracts were prepared from mouse hippocampi as described above. The protein concentration was determined using the Bio-Rad Protein Microassay and equal amounts of total hippocampal extracts were incubated in 25 mM MES Buffer, pH 7.0, 5 mM DTT, 2 mM EDTA and protease inhibitors + 1% Triton X-100 for 1 hour at 4°C. After incubation, samples were centrifuged at 100,000 *g* for 1 hour at 4°C (Optima Max Ultracentrifuge, Beckman Instruments). The resulting DRM pellet was re-suspended in Laemmli buffer to be processed directly by Western blot. DRM purity was confirmed by the presence of flotillin1 and the absence of transferring receptor 1.

### Precipitation of proteins with methanol and chloroform

Membrane proteins at low concentrations were recovered from solutions containing high concentration of detergents or salts through precipitation with methanol and chloroform as described by Wessel et al. [30]. The resulting pellet was re-suspended in PBS + 0.2% SDS or directly in Laemmli buffer for Western blot analysis.

### Western blotting

For each sample, equivalent amounts of protein were resolved by 10% or 12% SDS-polyacrylamide gel electrophoresis and transferred to a nitrocellulose membrane. The blocking step was performed in 5% milk in Tris-buffered saline with 0.1% Tween 20 (TBS-T) for 1 hour. Membranes were incubated for 2 hours at room temperature or overnight at 4°C, with the corresponding primary antibodies. Afterwards, blots were incubated with horseradish peroxidase-linked secondary antibodies (1:10,000; Zymed Laboratories, Invitrogen) and developed using chemiluminescence detection (ECL, Amersham). Images were taken using a Fujifilm LAS-3000 system and quantified with ImageJ 1.37v software (NIH, USA).

The following primary antibodies were used: D18, humanized monoclonal anti PrP (1:1,000; InPro Biotechnology, Inc, South San Francisco), anti-transferrin receptor1 (1:500; Invitrogen, Paisley, UK), mouse monoclonal anti PSD95 (1:100; Sigma), mouse monoclonal anti Flotillin1 (1:1,000; BD Transduction), mouse monoclonal anti Flotillin2 (1:1,000; BD Transduction), mouse monoclonal anti α-tubulin (1:10,000; Calbiochem), mouse monoclonal anti Vinculin (1:5,000; Sigma-Aldrich), anti β-actin HRP conjugated (1:25,000; Sigma-Aldrich).

### Preparation of functional synaptosomes

Hippocampal synaptosomes were isolated from C57BL/6J mice following the protocol from Nagy et al. [31]. Briefly, mouse hippocampi were dissected in ice, washed twice in cold PBS then homogenized in 5 mM HEPES, pH 7.4, 320 mM sucrose, 1 mM EDTA using a Dounce homogenizer with 10 strokes at 200-250 rpm. The homogenate was centrifuged at 3,000 *g* for 10 min at 4°C and the supernatant recovered and centrifuged again at 14,000 *g* for 12 min at 4°C. The resulting pellet was carefully re-suspended in Krebs-Ringer buffer (10 mM HEPES, pH 7.4, 140 mM NaCl, 5 mM KCl, 5 mM glucose, 1 mM EDTA), Percoll solution was added (final concentration 45% v/v) and the solution was mixed gently inverting the tube. After centrifugation at 13,000 *g* for 2 min at 4°C, the synaptosomal fraction was recovered at the surface of the flotation gradient and carefully re-suspended in Krebs-Ringer buffer. The pellet resulting from another centrifugation at 13,000 *g* for 30 sec at 4°C, representing the functional synaptosomal preparation, was re-suspended in an appropriate volume of Krebs-Ringer buffer. Synaptosomal purity was confirmed by the presence of synaptic marker PSD95 and the absence of transferrin receptor.

### Addition of sphingomyelin to functional synaptosomes

Synaptosomal preparations from the hippocampus of a single mouse were re-suspended in 400 µL of Krebs-Ringer buffer and split into two 1.5 mL Eppendorf tubes. Sphingomyelin (stock solution of 5 mg/mL in ethanol, Sigma-Aldrich) was added in one of the tubes, at a final concentration of 100 µg/mL; the same amount of pure ethanol was added in the control tube. Synaptosomes were incubated in a thermomixer at 300 rpm for 30 min at 37°C. Synaptosomes were centrifuged at 13,000 *g* for 30 sec at 4°C, washed once in Krebs-Ringer buffer and re-suspended in an appropriate volume of 25 mM MES Buffer, pH 7.0, 5 mM dithiothreitol, 2 mM EDTA containing protease inhibitors (Roche). Protein concentration was determined using the Bio-Rad Protein Microassay.

### Primary hippocampal cultures

Primary cultures were prepared from Wistar rat fetuses at embryonic day 18 (E18) as described in Kaech and Banker (2006) [32]. Animals were anaesthetized by intra peritoneal administration of a mixture of ketamine (75 mg/kg) and xylazine (10 mg/kg).

For biochemical analysis, 3-cm plastic dishes were coated with 0.1 mg/mL of poly-L-lysine (PLL), overnight at 37°C. Dissociated cells were plated at a density of 150,000 to 200,000 per dish in minimal essential medium with 10% Horse serum (MEM-Horse). After 5-24 hours, medium was replaced with minimal essential medium with N2 supplement (MEM-N2). After 5 days, medium was replaced again with Neurobasal medium with B27 supplement. Neurons were grown at 37°C and under 5% CO_2_.

For microscopy experiments, cells were plated on glass coverslips coated with 1 mg/mL of PLL, overnight at 37°C and small paraffin dots were placed on the edges of the coverslip. Six coverslips were placed in a 6-cm plastic dish and dissociated cells were plated at a density of 150,000 per dish in minimal essential medium with 10% Horse serum (MEM-Horse). After 5-24 hours coverslips were flipped in an astrocyte dish (neurons facing down) pre-incubated for 24 hours in minimal essential medium with N2 supplement (MEM-N2). Neurons were grown at 37°C and under 5% CO_2_.

Astrocytes were prepared from cerebral hemisphere of 1-day old (P1) Wistar rat pups. Astrocyte cultures take 2 weeks to be ready for use as feeder layer for neuronal cultures. Ideal confluence of astrocytes is around 50%.

### Immunolabeling of cultured neurons

Neurons grown on PLL-coated glass coverslips were fixed with 4% p-formaldehyde, permeabilized with 0.1% Triton X-100 and treated with blocking solution (2% fetal calf serum, 2% bovine serum albumin, and 0.2% fish skin gelatin) for 30 min at room temperature. Then, neurons were incubated with the primary antibodies for 60 min at room temperature. Finally, neurons were exposed to the corresponding secondary antibodies (1:1,000; Alexa Fluor) for 45 min. Images were taken using a Nikon confocal microscope.

### Surface immunolabeling of cultured neurons

Neurons grown on PLL-coated glass coverslips were incubated in their growth medium with the primary antibody of interest for 30 min at 37°C and under 5% CO_2_. Dynasore (Sigma) at a final concentration of 2.7 µL/mL was added to block endocytosis during incubation time. Neurons were washed twice in warm culture medium and twice in warm HBSS prior to fixation with 4% p-formaldehyde. Neurons were then treated with blocking solution (2% fetal calf serum, 2% bovine serum albumin, and 0.2% fish skin gelatin) for 30 min and subsequently exposed to the corresponding secondary antibody (1:1,000; Alexa Fluor) for 45 min.

After surface immunolabeling, neurons were permeabilized with 0.1% Triton X-100 and treated again with blocking solution for 30 min at room temperature. They were subsequently incubated with primary antibody for 60 min at room temperature, and exposed again to the corresponding secondary antibody (1:1,000; Alexa Fluor) for 45 min. Images were taken using a Nikon confocal microscope.

### Antibodies for immunolabeling

Recombinant anti-PrP^C^ humanized (HuM) Fab D18 was purchased from InPro Biotechnology, Inc, South San Francisco. D18 was used for immunolabeling to a final concentration of 10 µg/mL. HuM-D18 shows high affinity for PrP^C^, it has a large accessibility to its specific epitope — the region spanning residues 133-152 in the first alpha-helix of PrP^C^ — and binds the largest fraction of the cell-surface PrP^C^ population [33,34]. For co-immunolabeling experiments we used the following antibodies: MN7.51, mouse monoclonal anti Tau (1:10; previously described in Novak et al. [35]), rabbit polyclonal anti MAP2 (1:500; Santa Cruz), mouse monoclonal anti PSD95 (1:100; Sigma), mouse monoclonal anti Synaptophisin (1:100; SySy).

### Cell lines and cell culture

GT1 and ScGT1 cells were maintained in Dulbecco’s Modified Eagle’s Medium with 4.5 g/L glucose (DMEM) (GIBCO/Invitrogen), supplemented with 10% v/v fetal bovine serum (GIBCO/Invitrogen) and antibiotics (100 IU/mL penicillin and 100 mg/mL streptomycin) at 37°C in a humidified atmosphere with 5% CO_2_. All cell lines were kindly provided by Dr. P. Mellon (The Salk Institute, La Jolla, CA, USA) [36]. Scrapie cells were chronically infected with Rocky Mountain Lab (RML) prion strain according to published procedures.

### Cell culture treatment with sphingomyelin or fumonisin B1

Both GT1 and ScGT1 cells were split 2 days before treatment with SM or fumonisin B1 (FB1). On the second day after splitting, the medium was refreshed and either SM or FB1 was added. SM (stock solution of 10 mg/mL in ethanol, Sigma-Aldrich) was added at a final concentration of 100 µg/mL. The same amount of pure ethanol was added in the control culture. SM treatments lasted 30 minutes, 2 days and 7 days, respectively.

FB1 (1 mM stock solution in 20 mM Hepes, Sigma-Aldrich) was added at a final concentration of 25 µM. The same volume of Hepes buffer was added in the control culture. Also FB1 treatment was carried on for 2 days and 7 days, respectively. At the end of the treatment, proteins were extracted from cells in culture to determine total PrP levels or, alternatively, for proteinase K digestion.

### Proteinase K digestion assay

ScGT1 cells were washed twice in cold PBS, lysed in lysis buffer (10 mM Tris-HCl pH 8.0, 150 mM NaCl, 0.5% nonidet P-40 substitute, 0.5% deoxycholic acid sodium salt) and pelleted by centrifugation at 2,300 *g* for 5 min. The supernatant was collected and the total protein concentration was measured using bicinchoninic acid assay (Pierce). For the proteinase K digestion, 250 µg of protein was treated with 2.5 µg proteinase K (Roche) for 1 hour at 37°C. Digestion was stopped adding phenylmethyl sulphonyl fluoride at a final concentration of 2 mM. PrP was precipitated by ultracentrifugation at 100,000 *g* (Optima TL, Beckman) for 1 hour at 4°C. After centrifugation, the supernatant was discarded and the pellet was re-suspended in Laemmli buffer for Western blot analysis.

### Statistical analysis

Comparisons between groups were performed with the Student *t* test. Differences were considered significant when *p*<0.05. All data are expressed as mean value ± SD and the values of controls are adjusted to 1.

## Results and Discussion

### PrP^C^ expression in vivo

Previous works have analyzed PrP^C^ expression pattern in the brain of several animal species by means of different techniques [3,4]. These studies have focused mainly on PrP^C^ expression levels from early postnatal days to adulthood. We decided to extend our analysis to include the postnatal development of mouse brain, as well as adult and aging brains. In particular, we targeted the hippocampus because: (i) PrP^C^ is particularly abundant in this specific brain area [3,4]; and (ii) most PrP knockout (KO) mouse phenotypes are related to hippocampal abnormalities [37,38].

**Figure 1:**
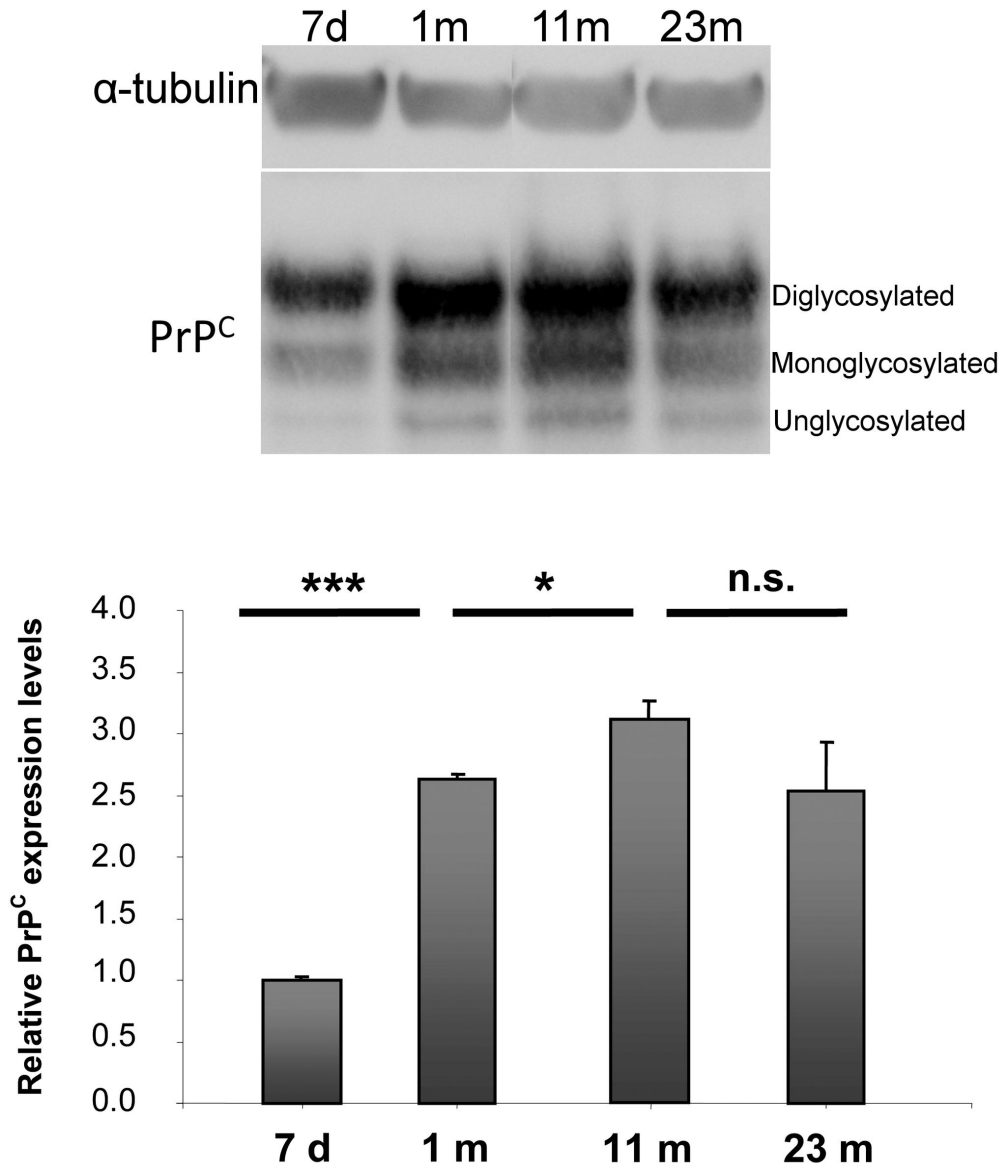
PrP^C^ expression levels in membrane extracts from mouse hippocampi at different developmental stages. Western blot analysis of equal amounts of protein from hippocampal membrane extracts (25 µg per lane) at the indicated ages. 7 d = 7 days old; 1 m = 1 month old; 11 m = 11 months old; 23 m = 23 months old. Each lane corresponds to a single animal. Antibodies used: D18 (1:1,000; InPro Biotechnology, Inc, South San Francisco), mouse monoclonal anti α-tubulin (1:10,000; Calbiochem). The three major PrP^C^ glycosylation forms are visible. Relative PrP^C^ expression levels were analyzed from 3 to 4 mice per time point. Each data point represents the relative protein level normalized over α-tubulin ± SD. Changes in band intensity were analyzed and quantified with ImageJ 1.37v software (NIH, USA) followed by comparison with ANOVA test for groups of mice at different ages. Differences were considered significant when *p*<0.05. PrP^C^ levels in hippocampal membrane from mice increased dramatically at the time of synaptogenesis (1 m), rose further during adulthood (11 m) and then remained at plateau during aging (23 m). n.s.: not significant, *: *p*<0.05, ***: *p*<0.001.

We analyzed PrP^C^ expression pattern at different stages of mouse brain development: early postnatal stage (day 7), completed synaptogenesis (1 month), full adulthood (9-10 months) and aging (23-24 months). Total membrane extracts from mouse hippocampi were analyzed by Western blotting, and the total amount of PrP^C^ was considered. Typical electrophoretic mobility of PrP^C^ and consequently its patterning in Western blotting is represented by 3 bands. The most prominent band is centered at 36 kDa, a second band at 33 kDa (in some cases visible as a double band, depending on the degree of maturation of the glycolic chain) and a less intense one at 25 kDa. Each band represents a different glycosylation form of the protein. In our analysis, we considered the total amount of PrP^C^. In agreement with previous works [3,4], we observed that PrP^C^ level in hippocampal membrane protein extracts from wild-type mice increased at the time of synaptogenesis (1 month), peaked during adulthood (9-10 months) and tended to remain constant during aging (20-24 months) (Figure 1).

### PrP^C^ compartmentalization on plasma membrane during aging

Previous studies have shown that PrP^C^ is associated for much of its life cycle with lipid rafts, membrane domains rich in cholesterol and glycosphingolipids [39,40]. DRMs are defined as that part of the cellular membrane that is insoluble in cold non-ionic detergents. Although from a biochemical standpoint DRMs cannot be considered to correspond to *in vivo* rafts, they may be used to assign raft-association when changes in DRM composition are induced by biochemically/physiologically relevant events.

**Figure 2:**
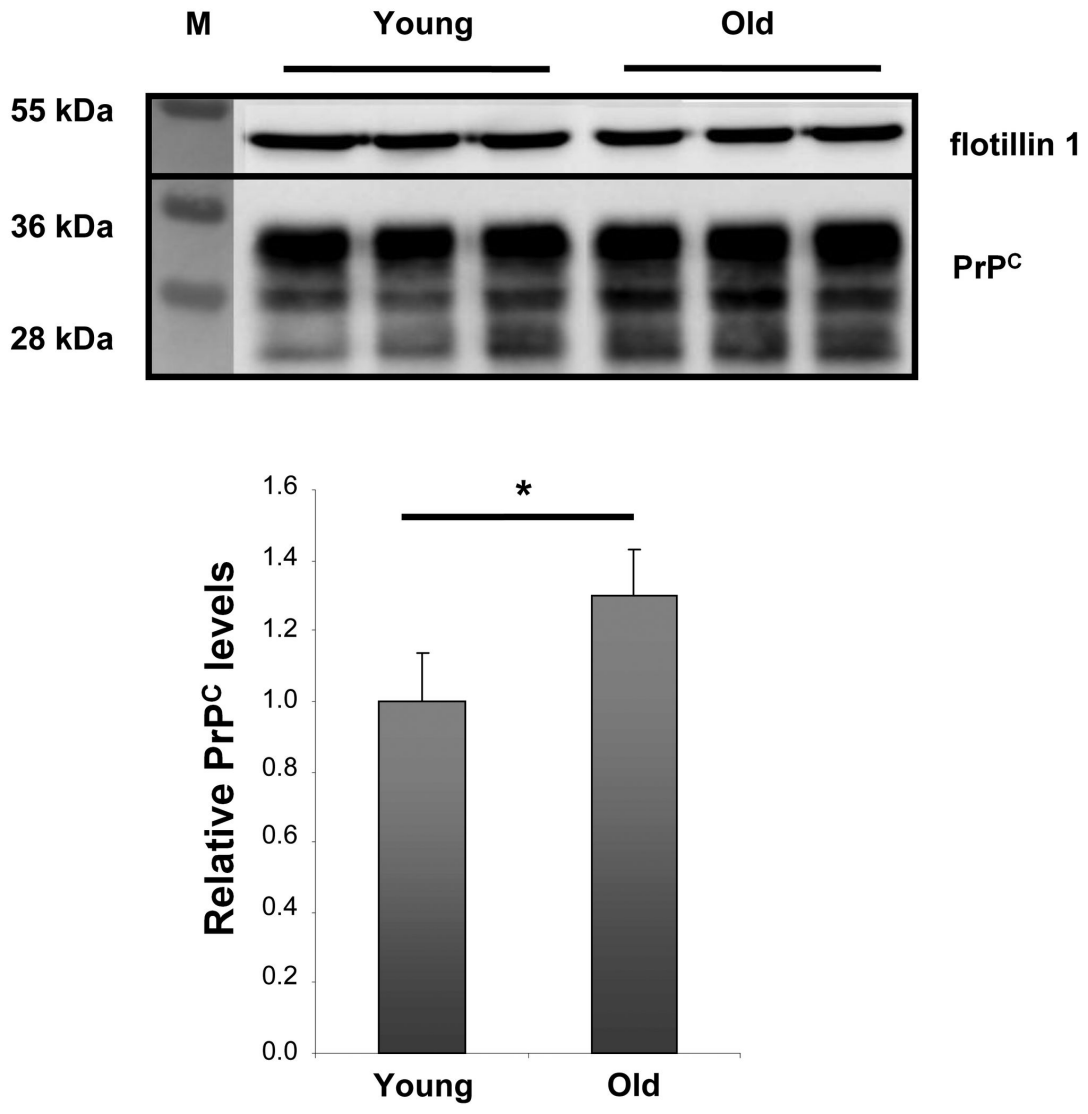
PrP^C^ in DRM preparation of young (3-4 months) vs. old (20-21 months) mice. Western blot analysis of DRMs prepared from equal amounts of total hippocampal protein extracts (100 µg of total protein) at the indicated ages. Young = 3-4 months old; old = 21-22 months old. Each lane corresponds to a single animal. Antibodies used: D18 (1:1,000; InPro Biotechnology, Inc, South San Francisco), mouse monoclonal anti flotillin1 (1:1,000; BD Biosciences). Relative PrP^C^ amounts from 3 mice per time point were analyzed. Each data point represents the relative protein level normalized over flotillin1 ± SD.

It has been shown that the aging process is accompanied by loss of cholesterol and increase in sphingolipids in mouse hippocampi. Martin and colleagues showed a decrease in cholesterol content in mouse hippocampus during development [21,22] and the lowest levels were detected in hippocampal membranes of aging animals (21 months old) [21,22,25]. Moreover, cholesterol decrease has been shown to parallel an increase in SM, which reaches its highest levels in 23-month-old animals [23]. Thus, we decided to investigate whether changes in the two major lipid components of rafts have any influence on PrP^C^ association with DRMs. Comparing DRMs from hippocampi of young adult mice (3-4 months old) with those of aging animals (20-21 months old), we found that PrP^C^ levels in DRMs increased by around 30% during aging (Figure 2). No difference in total protein content was found (Figure S1). To enhance the confidence of our results, the purity of DRM preparations as well as the amount of PrP^C^ associated with detergent-soluble membranes (DSMs) were also determined (Figures S2 and S3). Confirming previous findings, PrP^C^ levels in DSMs were found 30% lower in old mice than in young animals, although this change cannot be considered statistically significant.

Even though PrP^C^ expression levels tend to remain constant in the hippocampus during aging, we observed that changes in the cholesterol/SM ratio in this brain area are accompanied by an enrichment of PrP^C^ in DRMs.

### PrP^C^ compartmentalization after membrane lipid alteration

In order to test the cause-effect relationship between lipid changes and PrP^C^ enrichment in DRM preparations, we analyzed PrP^C^ compartmentalization in systems where the lipid ratio is different from the physiological state and rather resembles that seen during aging. We investigated systems in which SM levels are modified or can be easily manipulated.

**Figure 3:**
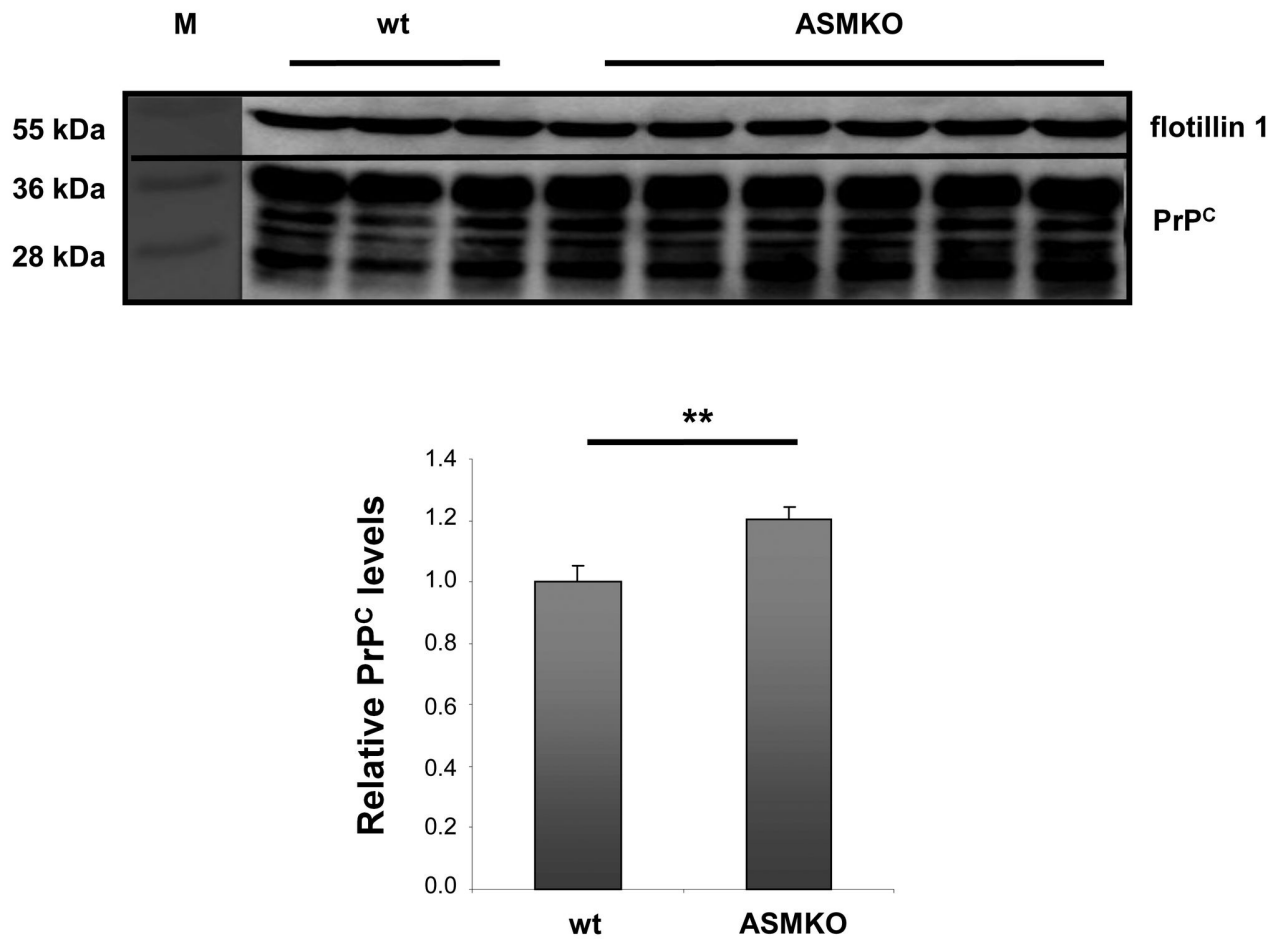
PrP^C^ in DRMs from hippocampal membrane of young wild-type mice compared with age-matched ASMKO mice. Western blot analysis of DRMs prepared from equal amounts of hippocampal extracts (100 µg of protein) from young (4-5 months old) wild-type and ASMKO mice. Each lane corresponds to a single animal. Antibodies used: D18 (1:1,000; InPro Biotechnology, Inc, South San Francisco), mouse monoclonal anti flotillin1 (1:1,000; BD Biosciences). Quantification of relative PrP^C^ amounts from 3 control mice and 6 ASMKO mice. Each data point represents the relative PrP level normalized over flotillin1 ± SD. PrP^C^ levels are 20% higher in ASMKO mice compared to age-matched wild-type mice. **: *p*<0.01.

First we looked at PrP^C^ compartmentalization in hippocampi from ASMKO mice. These mice represent an animal model for Niemann–Pick disease type A [41]. This is a suitable model for our studies because the ASMKO neurons show anomalously high SM levels at the plasma membrane caused by the absence of the ASM enzyme [42]. We compared PrP^C^ levels in DRMs from hippocampi of wild-type and ASMKO mouse littermates. We performed Western blotting analysis on DRM preparations from hippocampi from 5-month-old animals. At this developmental stage ASMKO mice are still asymptomatic but their membranes are already enriched in SM (Figure S4). In agreement with the hypothesized cause-effect relation, PrP^C^ was highly enriched in DRMs derived from ASMKOs. PrP^C^ levels in DRMs of ASMKO mice were 30% higher than in wild-type littermates (Figure 3), though total protein content resulted unchanged (Figure S5). No difference in PrP^C^ levels was detected in DSMs (Figure S6).

**Figure 4:**
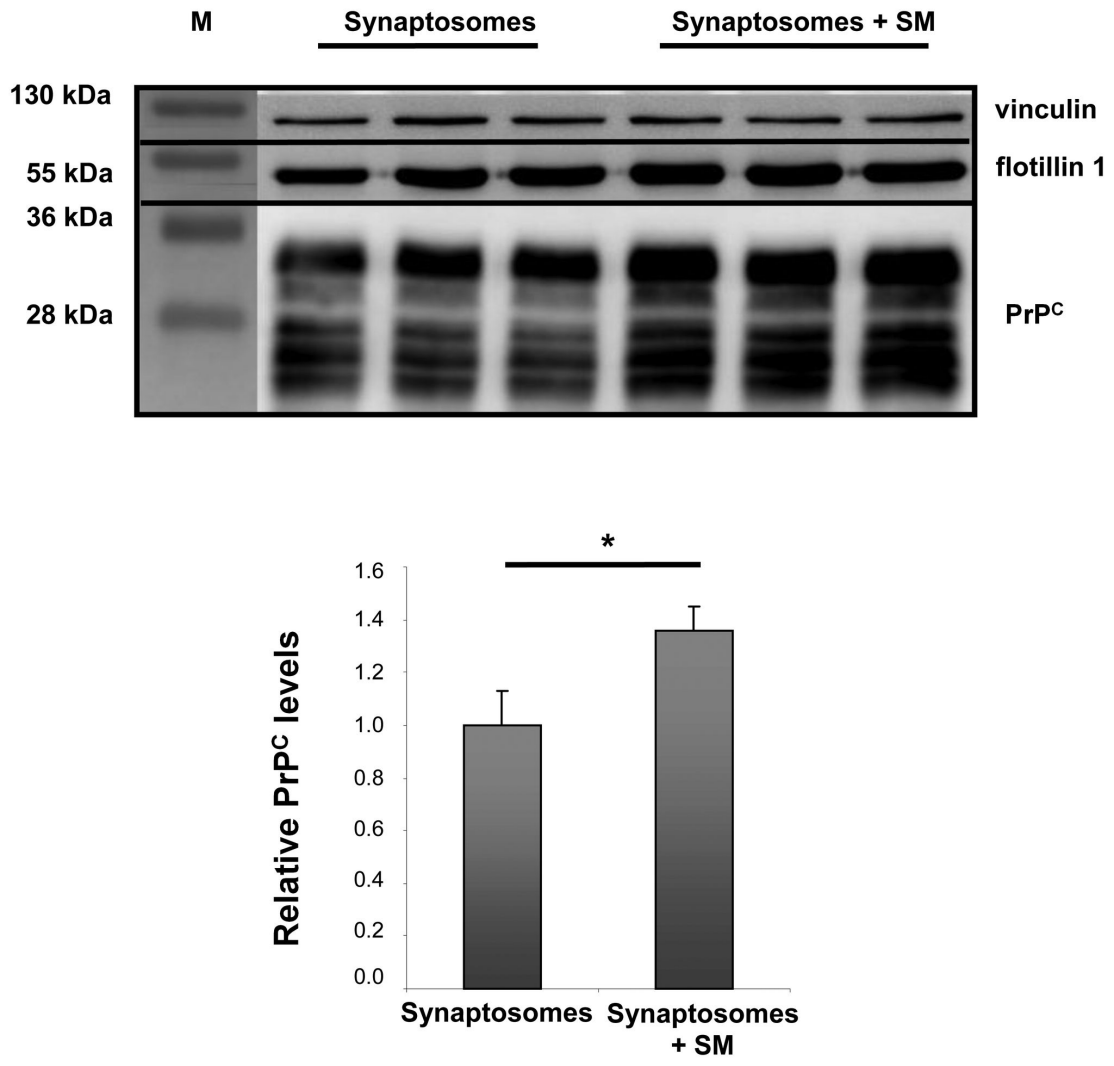
PrP^C^ in DRMs from functional synaptosomes treated with sphingomyelin. Western blot analysis of DRMs prepared from synaptosomes treated with sphingomyelin for 30 min (100 µg/mL) and relative controls. Antibodies used: D18 (1:1,000; InPro Biotechnology, Inc, South San Francisco), mouse monoclonal anti flotillin1 (1:1,000; BD Biosciences), mouse monoclonal anti vinculin (1:5,000; Sigma-Aldrich). Relative PrP^C^ amounts from 3 preparations per condition. Each data point represents the relative PrP level normalized over vinculin ± SD. Sphingomyelin treatment determined about 30% increase in PrP^C^ levels in DRMs. *: *p*<0.05.

Several immuno-electron microscopy studies have shown that PrP^C^ is localized in synaptic boutons [43]. Therefore, to further confirm a cause-effect relationship, we measured PrP^C^ levels in DRMs prepared from functional synaptosomes exposed to SM. Purity of synaptosomal preparations was confirmed by the presence of synaptic marker PSD95 and the absence of transferrin receptor (Figure S7). Fresh functional synaptosomes were incubated for 30 min at 37°C in the presence of SM (100 µg/mL) and subsequently isolated, and DRMs were analyzed. This acute treatment, as expected, led to an increase in PrP^C^ levels in DRMs, but differently from what we had seen in previous experiments, it also increased flotillin1, a protein used as marker for DRMs, employed to normalize DRM blots. Considering that the treatment was performed on the same amounts of synaptosome preparations and the same synaptosomal preparation was compared with and without SM treatment, we performed also a normalization of PrP content over vinculin, a cytoskeleton-associated protein. This allowed us to record a 35% increase in PrP^C^ levels in DRMs with respect to controls after SM addition (Figure 4).

We therefore showed that an increase in SM content in cellular membranes determines accumulation of PrP^C^ in DRMs. This accumulation could be due to the different affinity between the GPI-anchor and SM enriched lipid rafts with respect to cholesterol-enriched lipid rafts.

### PrP^C^ expression and localization in vitro

**Figure 5:**
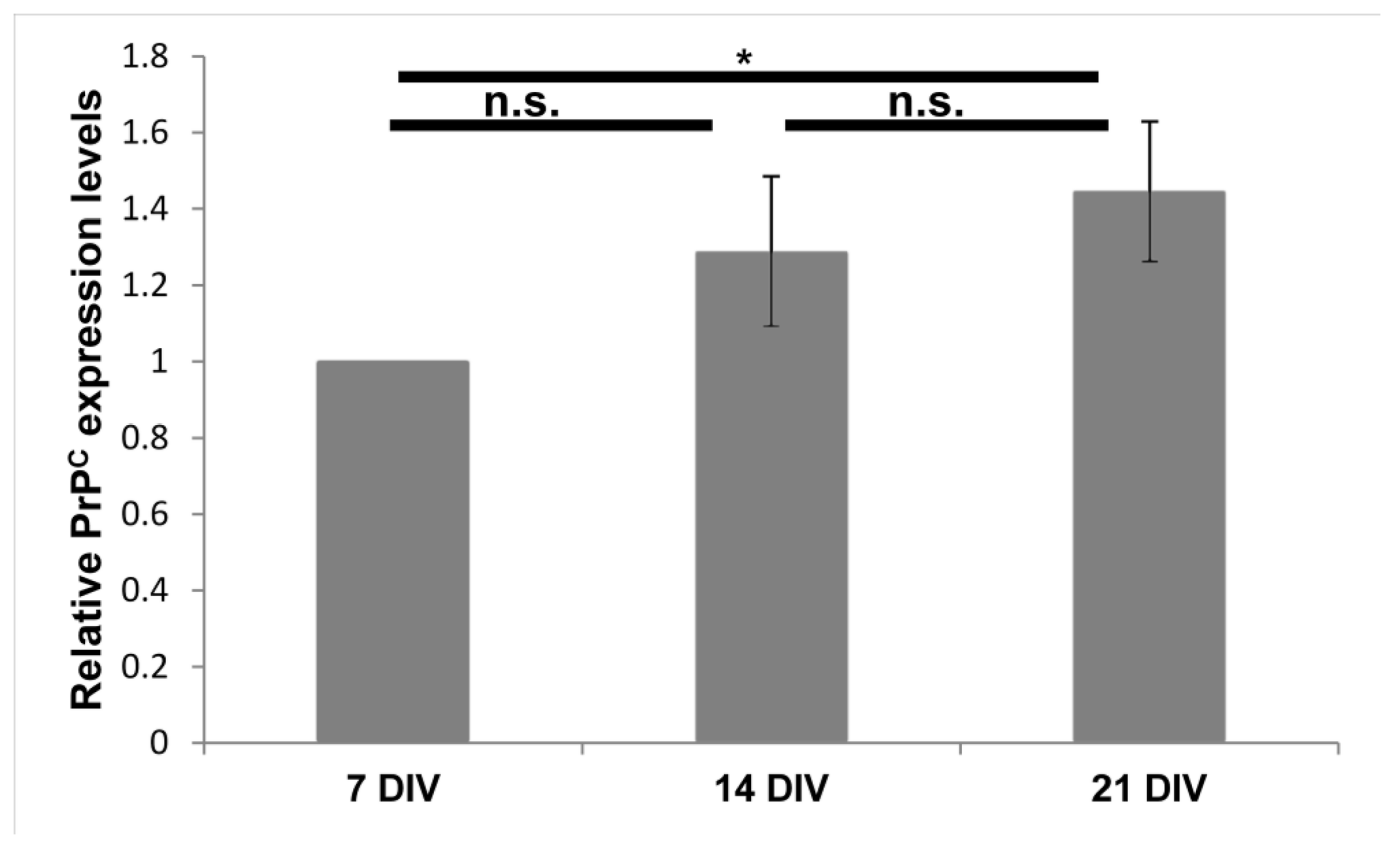
PrP^C^ expression levels in total protein extracts from primary neurons at different developmental stages. Relative PrP^C^ amount from 3 cultures per time point. Each data point represents the mean protein level normalized over tubuline ± SD. n.s.: not significant, *: p<0.05.

The expression of PrP^C^ and its cellular localization were further investigated in primary rat hippocampal neurons *in vitro*. We analyzed by Western blotting PrP^C^ expression levels at different developmental stages: before synaptogenesis (7 DIV), after synaptogenesis (14 DIV) and in mature to aging neurons (21 DIV) (Figure 5). The results confirmed PrP^C^ up-regulation over time.

**Figure 6:**
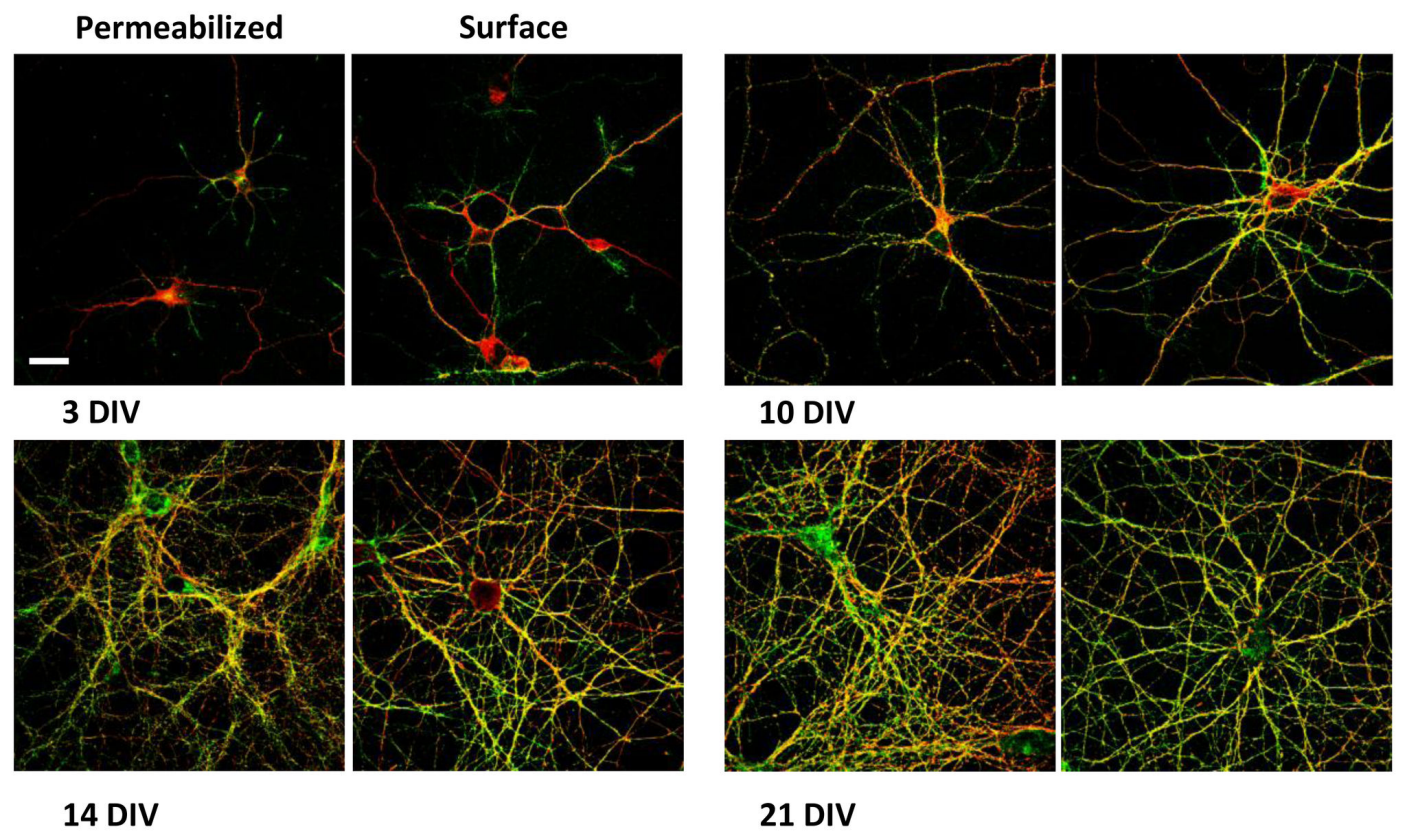
PrP^C^ co-immunolabeling with Tau. Confocal images of hippocampal primary neurons at different developmental stages. Normal (left) and surface (right) immunolabeling of PrP^C^ (green) coupled with Tau staining (red). Antibodies used: D18 (10 µg/mL and 20 µg/mL in surface immunolabeling; InPro Biotechnology, Inc, South San Francisco), MN7.51, mouse monoclonal anti Tau (1:10; previously described in Novak et al., 1991). Scale bar = 10 µm.

**Figure 7:**
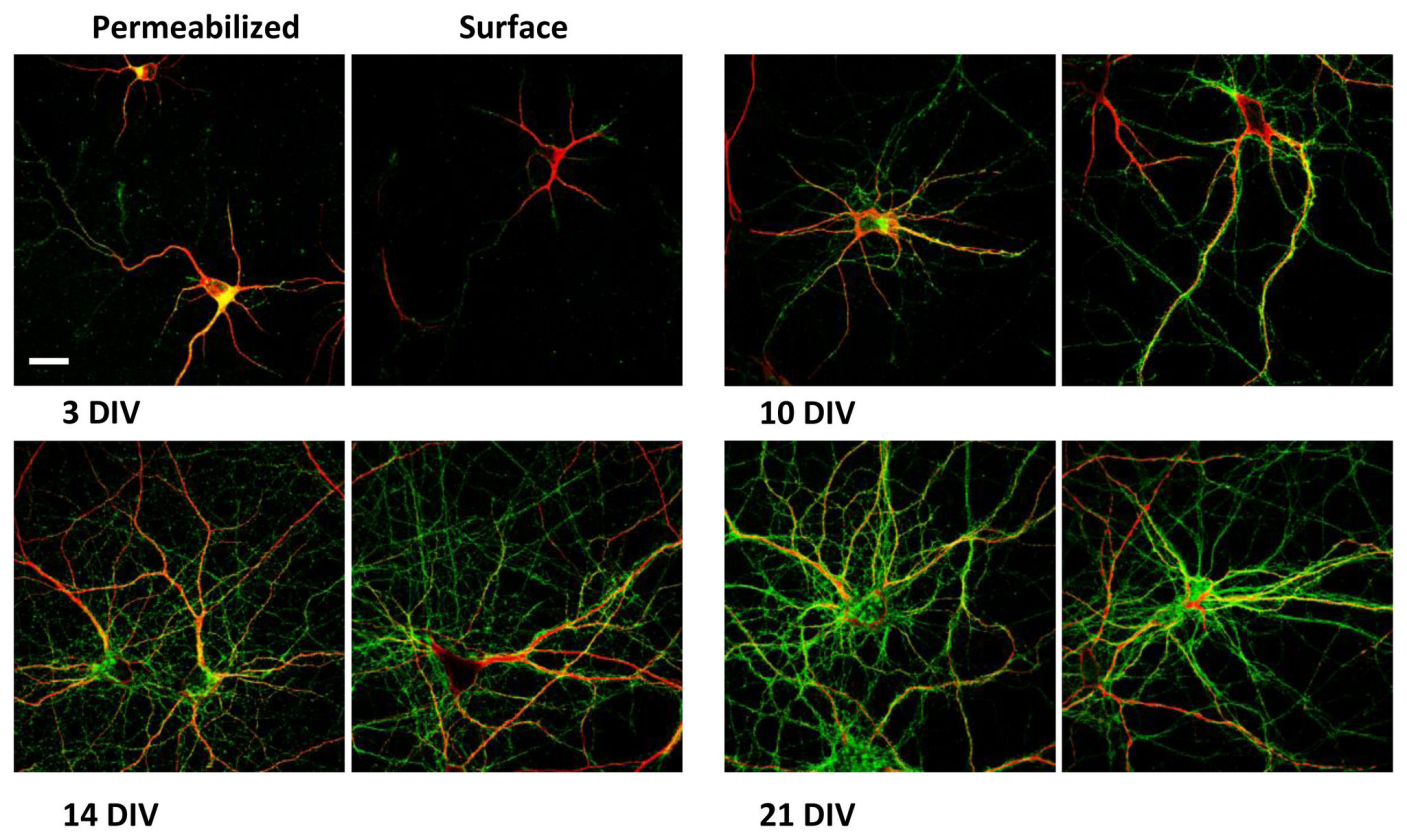
PrP^C^ co-immunolabeling with MAP2. Confocal images of hippocampal primary neurons at different developmental stages. Normal (left) and surface (right) immunolabeling of PrP^C^ (green) coupled with MAP2 staining (red). Antibodies used: D18 (10 µg/mL and 20 µg/mL in surface immunolabeling InPro Biotechnology, Inc, South San Francisco), rabbit polyclonal anti MAP2 (1:500; Santa Cruz). Scale bar = 10 µm.

**Figure 8:**
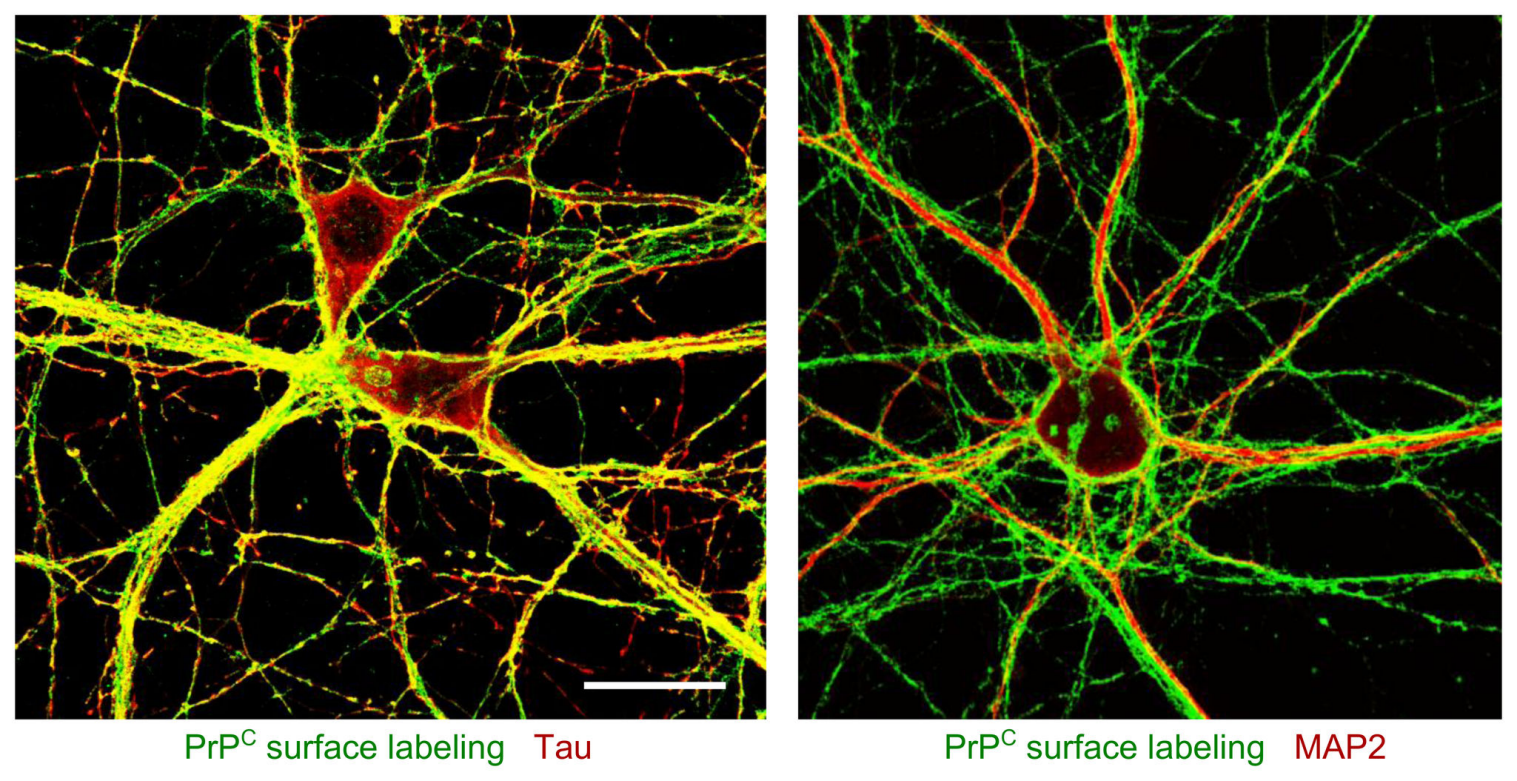
PrP^C^ co-immunolabeling with Tau and MAP2. Confocal images of 21 DIV hippocampal primary neurons. Surface immunolabeling of PrP^C^ (green) coupled with Tau staining (red) (left) or MAP2 staining (red) (right). Antibodies used: see figures 6 and 7. Scale bar = 10 µm.

To determine PrP^C^ localization in the different cell compartments at different developmental stages (from 3 DIV to 21 DIV) immunocytochemical experiments were performed on cultured hippocampal neurons. This involved normal and surface immunolabeling of PrP^C^ coupled with different cell markers (MAP2, Tau, PSD95 and synaptophysin). At early stages of development, PrP^C^ is present in all neurites, whereas in mature neurons it is localized only in axons (Figures 6 and 7). In mature neurons, indeed, PrP^C^ co-localizes with neurites enriched in Tau protein and it does not co-localize with the dendritic marker MAP2 (Figure 8). We also observed that the pool of PrP^C^ in mature axons is mainly restricted to the surface, whereas in immature neurons most of the protein is still localized in the internal cellular compartments undergoing sorting.

**Figure 9:**
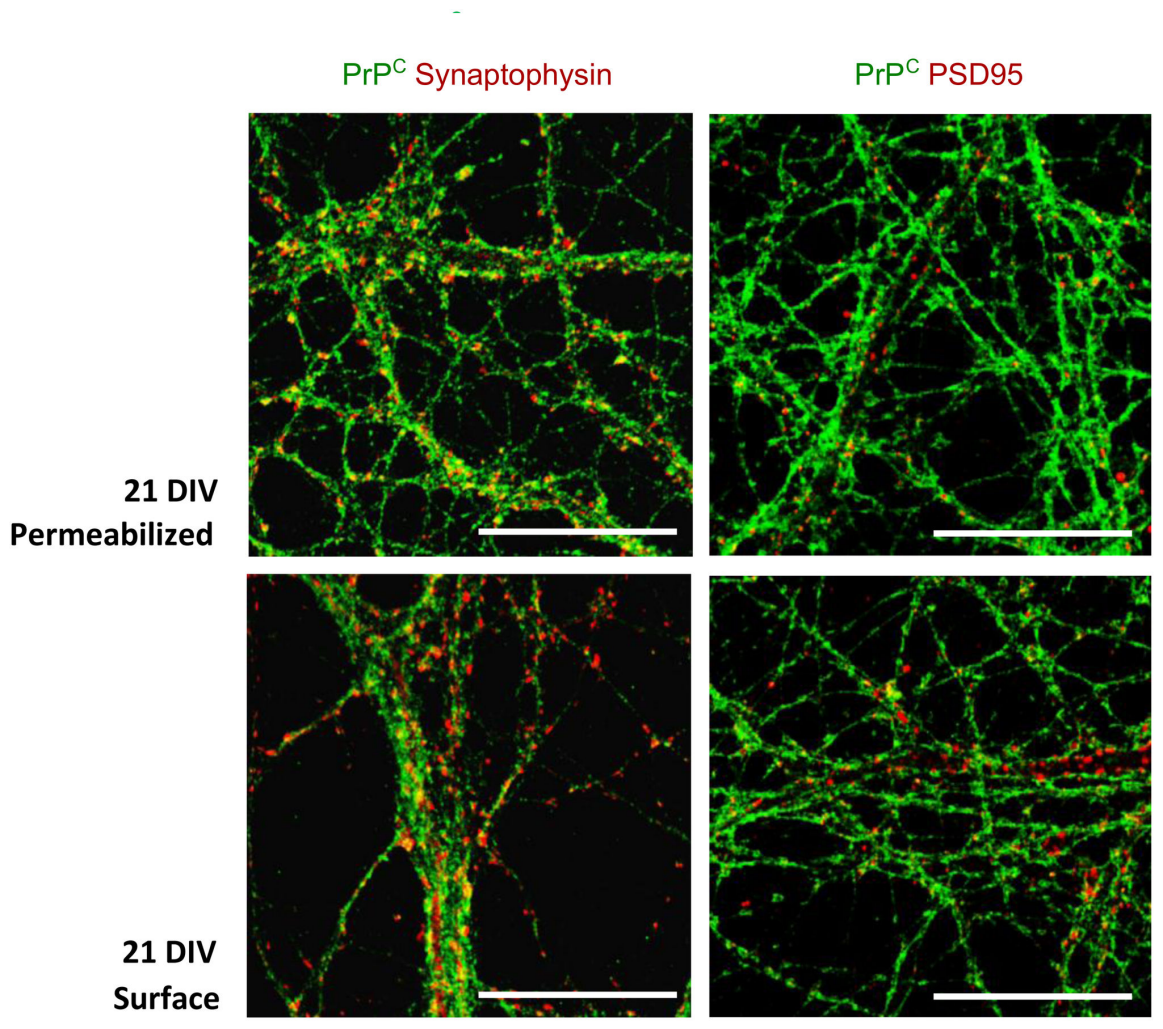
PrP^C^ co-immunolabeling with synaptophysin (left) and PSD95 (right). Confocal images of fully developed hippocampal primary neurons (21 DIV). Normal (above) and surface immunolabeling (below) of PrP^C^ (green) coupled with synaptophysin and PSD95 (red). Antibodies used: D18 (10 µg/mL and 20 µg/mL in surface immunolabeling InPro Biotechnology, Inc, South San Francisco), mouse monoclonal anti Synaptophisin (1:100; SySy), mouse monoclonal anti PSD95 (1:100; Sigma). Scale bar = 10 µm.

To evaluate the pre/postsynaptic distribution of PrP^C^, we stained mature neurons (21 DIV) with different synaptic markers like synaptophysin (pre-synaptic marker) and PSD95 (post-synaptic marker) (Figure 9). A partial co-localization was observed with synaptophysin, but not with PSD95.

### Do changes in PrP^C^ compartmentalization affect PrP^Sc^ formation?

We observed that changes in the cholesterol/SM ratio physiologically occurring during aging are accompanied by an accumulation of PrP^C^ in DRMs.

Several lines of evidence suggest that the conversion of PrP^C^ into PrP^Sc^ can be influenced by lipid raft composition [12,15]. This phenomenon prompted us to investigate the effect of the changes in PrP^C^ compartmentalization on prion formation. Recently, several cell systems permissive to the replication of different mouse-adapted prion strains have been developed [44]. To investigate how membrane modifications in aging neurons affect prion formation, we used an immortalized murine cell line derived from hypothalamic cells either uninfected or chronically infected with RML prion strain (GT1 cells and ScGT1 cells hereafter, respectively). GT1 cells derive from central nervous system (CNS) neurons, and they are one of the best murine models available to investigate neurons *in vitro*.

We manipulated the sphingolipid content in ScGT1 cells and determined the effects on the formation of protease-resistant PrP^Sc^, a biochemical biomarker of prion infection. We analyzed protease-resistant PrP^Sc^ formation in cells treated either with SM or FB1, an inhibitor of sphingolipid biosynthesis.

In ScGT1 cells treated with SM (100 µg/mL) for 2 and 7 days, respectively, total PrP and protease-resistant PrP^Sc^ levels remained unchanged (Figure S8). To investigate the effects of SM treatment on PrP^C^ compartmentalization, we treated non-infected GT1 cells with SM for 30 minutes, 2 days and 7 days, respectively (Figure S9). No significant changes in PrP^C^ compartmentalization were observed, though the 30-minute treatment resulted in a slight increase of PrP^C^ in DRM preparations. This time window does not allow for the detection of any effect on protease-resistant PrP^Sc^ formation because it is generally necessary to treat cells for 2 days or longer to appreciate the effect of any drug on prion formation.

**Figure 10:**
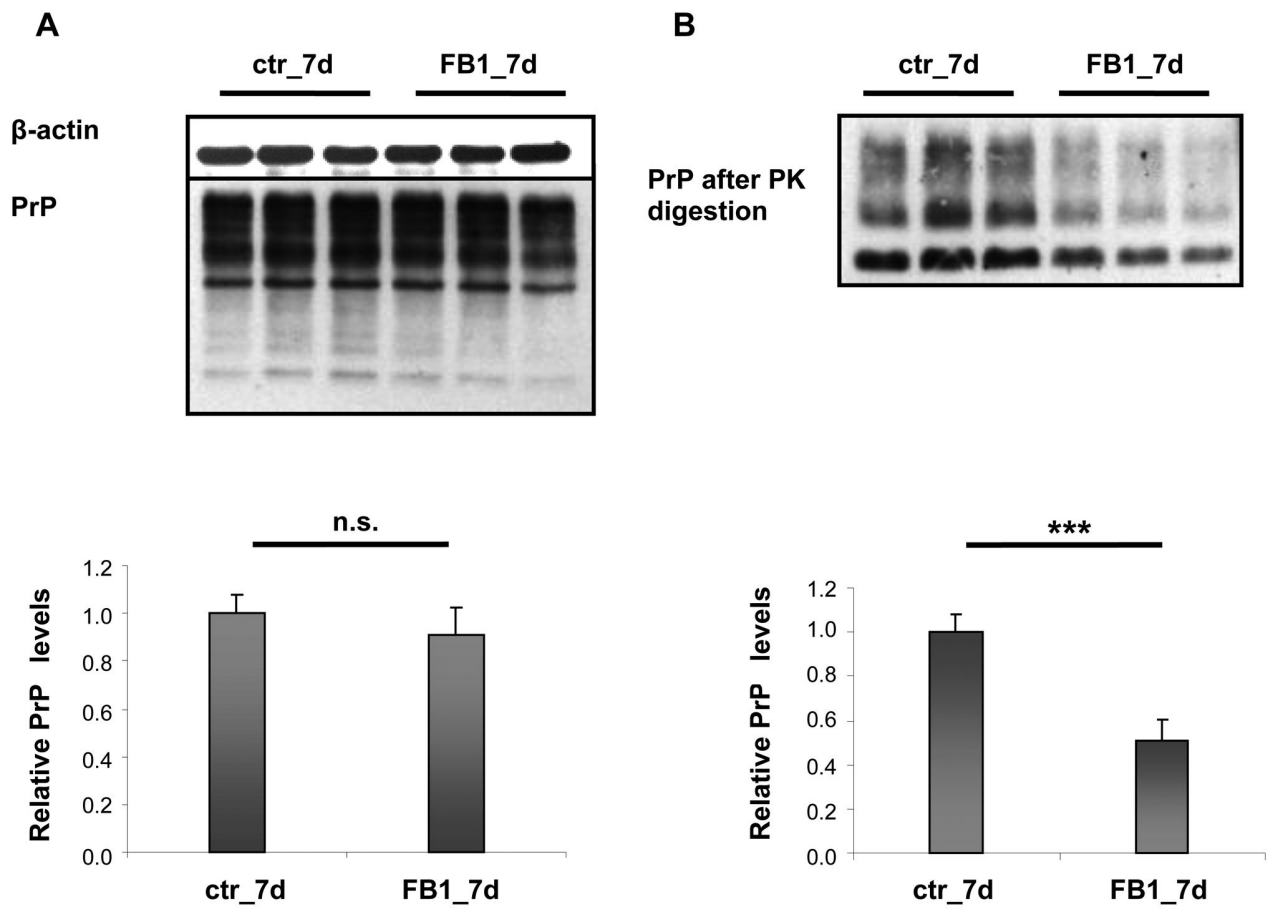
Total PrP and PK-resistant PrP in ScGT1 cells after treatment with FB1. ScGT1 cells were treated for 7 days (7d) with FB1 (25 µM). A) Western blot analysis of equal amounts of protein from ScGT1 cells (25 µg per lane). Antibodies used: D18 (1:1,000; InPro Biotechnology, Inc, South San Francisco), mouse monoclonal anti β-actin (1:25,000; Sigma-Aldrich). Each data point represents the mean protein level normalized over β-actin ± SD. B) Western blot analysis of equal amounts of protein from ScGT1 cells (250 µg per lane) after PK digestion. Antibodies used: D18 (1:1,000; InPro Biotechnology, Inc, South San Francisco) and mouse monoclonal anti β-actin (Sigma). Each data point represents the mean protein level normalized over total PrP ± SD. No significant changes in PrP levels were detected in the total protein extracts or in protease-resistant PrP after sphingomyelin treatment. FB1 treatment did not affect total PrP levels while protease-resistant PrP decreased to 50% compared to control cultures. n.s.: not significant, ***: *p*<0.001.

In order to relate changes in sphingolipid levels with the formation of protease-resistant PrP^Sc^, we treated cells with FB1, as it inhibits the biosynthesis of sphingolipids [45,46]. Cells treated with 25 µM FB1 for 2 days did not show any changes in either total PrP or protease-resistant PrP^Sc^ (data not shown). On the other hand, a longer treatment (7 days) again did not affect total protein levels but significantly reduced protease-resistant PrP^Sc^ to 50% compared to controls (Figure 10). Interestingly, our findings contrast with the results obtained by Naslavsky and colleagues using ScN2a cells [15]. This controversial result may be due to the differences between the cell lines used. GT1 cells are immortalized CNS neurons and represent a model for studying changes occurring in neurons *in vitro*, whereas N2a cells are cancerous cells from the peripheral nervous system, with different characteristics compared to CNS neurons. Because prion diseases mainly affect the brain, we decided to use an *in vitro* model that mimics CNS neurons as closely as possible

## Conclusions

It has been shown that brain aging is accompanied by changes in the cholesterol/sphingolipids ratio, thus influencing several cellular pathways [21–25]. In particular, a moderate loss of brain cholesterol and an increase in sphingolipids with age have been described in several studies [21–25]. Cholesterol and sphingolipids are the major constituents of lipid rafts, which are specialized membrane microdomains functioning as intracellular signaling platforms. Alterations in lipid raft composition were found in several degenerative disorders such as atherosclerosis, diabetes, cancer, muscular dystrophy and neurodegenerative disorders such as Alzheimer’s disease.

In prion diseases, the sporadic forms account for approximately 85% of all cases, mostly occurring during aging. Being PrP^C^ a GPI-anchored protein, it is associated with lipid rafts, and therefore changes in lipid raft composition during aging may greatly alter PrP^C^ compartmentalization. In turn, this change in localization may have consequences with regard to PrP^C^ physiological function and its propensity to be converted into prions.

In this study, we first analyzed PrP^C^ expression levels in both murine hippocampi and rat neurons in culture at different developmental stages. It has been established that the expression of PrP^C^ increases during the initial postnatal weeks up to the synaptogenic process completion, and it remains stable at plateau during adulthood. We found a slight decrease in PrP^C^ expression levels in old mice but this difference was not statistically significant. Investigations on primary rat hippocampal neurons *in vitro* confirmed a similar trend. Interestingly, a recent investigation found that PrP^C^ expression levels were reduced in the hippocampus of humans during aging and in patients with sporadic Alzheimer’s disease [47]. The discrepancy in PrP^C^ expression levels found between rodents and humans may reflect the different lifespan between these species.

In this study we found that the compartmentalization of PrP^C^ was affected by lipid changes occurring at the neuronal membrane during aging. Indeed, we observed that the decrease in cholesterol and the increase in SM that physiologically occur in lipid rafts of hippocampal neurons *in vivo* were accompanied by an enrichment of PrP^C^ in DRMs and its reduction in DSMs.

Using model systems that were responsive to lipid manipulations allowed us to confirm a cause-effect relationship between rising SM in membranes and the accumulation of PrP^C^ in DRMs.

The molecular mechanism by which PrP^C^ is converted to PrP^Sc^ relies on the direct binding between the two molecules. An enrichment of PrP^C^ in DRMs may favor this pathological process. We therefore investigated how the physiological lipid changes at the membrane of aging neurons affect prion formation.

In our model (ScGT1) we showed that a reduction in sphingolipid levels, which mimics a juvenile condition, inhibits the formation of prions. This suggests an involvement of PrP-lipid environment in prion formation and consequently in the onset or development of sporadic prion diseases. Reduction in cholesterol and the concomitant increase in SM trigger the enrichment of PrP^C^ in lipid rafts, thus favoring the likelihood of inter-molecular contacts, and in the presence of PrP^Sc^, these changes lead to its pathological conversion.

Altogether, this is the first attempt to correlate membrane lipid content, PrP^C^ localization and prion formation at different stages of development, adulthood and later stages of life. Our results indicate that during aging, at least in murine models, PrP^C^ tends to accumulate in DRMs, a process that may lead to higher susceptibility to prion conversion. Although additional experiments are needed to confirm that a similar mechanism occurs in other mammals, our results indicate a plausible molecular basis for a higher incidence of sporadic prion disorders in aging animals and humans.

Our findings also represent an interesting link between aging, PrP^C^ localization, prion diseases and Alzheimer’s disease. PrP^C^ has been shown to mediate Aβ oligomer toxicity acting as a high affinity receptor [48]. PrP^C^-Aβ oligomers complex formation leads to inhibition of long-term potentiation and to memory impairments. Disruption of lipid rafts significantly reduces the cell surface binding of Aβ oligomers, thus preventing their toxicity [49]. Therefore, the enrichment of PrP^C^ into DRMs during aging, as a consequence of changes in lipid raft composition, may increase susceptibility to prion conversion as well as the binding of Aβ oligomers on cell surface and their toxicity.

## Supporting Information

Figure S1 **Total protein content in DRMs of hippocampi from young and aging mice.** Comparison of total protein levels in DRMs from hippocampi of young adult mice (3-4 months old) with those of aging animals (20-21 months old).
(TIF)

Figure S2 **Purity of DRM preparations.** Western blot analysis of DRM preparations. Antibodies used: anti-transferrin receptor1 (1:500; Invitrogen, Paisley, UK), mouse monoclonal anti Flotillin1 (1:1,000; BD Transduction), D18, humanized monoclonal anti PrP (1:1,000; InPro Biotechnology, Inc, South San Francisco).
(TIF)

Figure S3 **PrP^C^ in DSM preparation of young (3-4 months) vs. old (25 months) mice.** Western blot analysis of DSMs prepared from equal amounts of total hippocampal protein extracts at the indicated ages. Young = 3-4 months old; old = 25 months old. Antibodies used: D18 (1:1,000; InPro Biotechnology, Inc, South San Francisco). Relative PrP^C^ amounts from 3 mice per time point were analyzed. Each data point represents the relative protein level normalized over protein loading ± SD.
(TIF)

Figure S4 **Sphingomyelin in wild-type and ASMKO mice.** Comparison of sphingomyelin in 5-month-old wild-type and ASMKO mice. *: p<0.05.
(TIF)

Figure S5 **Total protein content in DRMs of hippocampi from young and ASMKO mice.** Comparison of total protein levels in DRMs from hippocampi of young adult mice (5 months old) with those of ASMKO littermates.
(TIF)

Figure S6 **PrP^C^ in DSMs from hippocampal membrane of young wild-type mice compared with age-matched ASMKO mice.** Western blot analysis of DSMs prepared from equal amounts of hippocampal extracts from young (4-5 months old) wild-type and ASMKO mice. Antibodies used: D18 (1:1,000; InPro Biotechnology, Inc, South San Francisco). Quantification of relative PrP^C^ amounts from 3 control mice and 3 ASMKO mice. Each data point represents the relative PrP^C^ level normalized over protein loading ± SD.
(TIF)

Figure S7 **Purity of synaptosomal preparations.** Western blot analysis of synaptosomal preparations. Antibodies used: PSD95 (1:100; Sigma), anti-Transferrin receptor (1:500; 13-6800 Invitrogen, Paisley, UK). *Total* indicates total homogenate, and *Syn* indicates synaptosomes.
(TIF)

Figure S8 **Total PrP and PK-resistant PrP in ScGT1 cells after treatment with SM.** ScGT1 cells were treated for 7 days (7d) with sphingomyelin (100 µg/mL). A) Western blot analysis of equal amounts of protein from ScGT1 cells (25 µg per lane). Antibodies used: D18 (1:1,000; InPro Biotechnology, Inc, South San Francisco), mouse monoclonal anti β-actin (1:25,000; Sigma-Aldrich). Each data point represents the mean protein level normalized over β-actin ± SD. B) Western blot analysis of equal amounts of protein from ScGT1 cells (250 µg per lane) after PK digestion. Antibodies used: D18 (1:1,000; InPro Biotechnology, Inc, South San Francisco) and mouse monoclonal anti β-actin (Sigma). Each data point represents the mean protein level normalized over total PrP ± SD. No significant changes in PrP levels were detected in the total protein extracts or in protease-resistant PrP after sphingomyelin treatment.
n.s.: not significant.
(TIF)

Figure S9 **PrP^C^ in DRMs from GT1 cells treated with sphingomyelin.** Western blot analysis of DRMs prepared from equal amounts of protein (150 µg of total protein) from GT1 cells treated with 100 µg/ml of sphingomyelin. A) GT1 cells treated for 2 days (2d). B) GT1 cells treated for 7 days (7d). C) GT1 cells treated for 30 minutes (30m). Antibodies used: D18 (1:1,000; InPro Biotechnology, Inc, South San Francisco), mouse monoclonal anti flotillin2 (1:1,000; BD Biosciences). Each data point represents the mean protein level normalized over flotillin2 ± SD. No significant changes in PrP levels could be detected in the DRMs after sphingomyelin long treatment (2d or 7d). After short treatment (30m) PrP showed a tendency to increase, although not significantly.
n.s.: not significant.
(TIF)
