# Supplementary figures and images for: Prion Protein Accumulation in Lipid Rafts of Mouse Aging Brain
Source: PLoS One. 2013 Sep 10;8(9):e74244. doi: 10.1371/journal.pone.0074244 (PMC3769255; doi:10.1371/journal.pone.0074244)

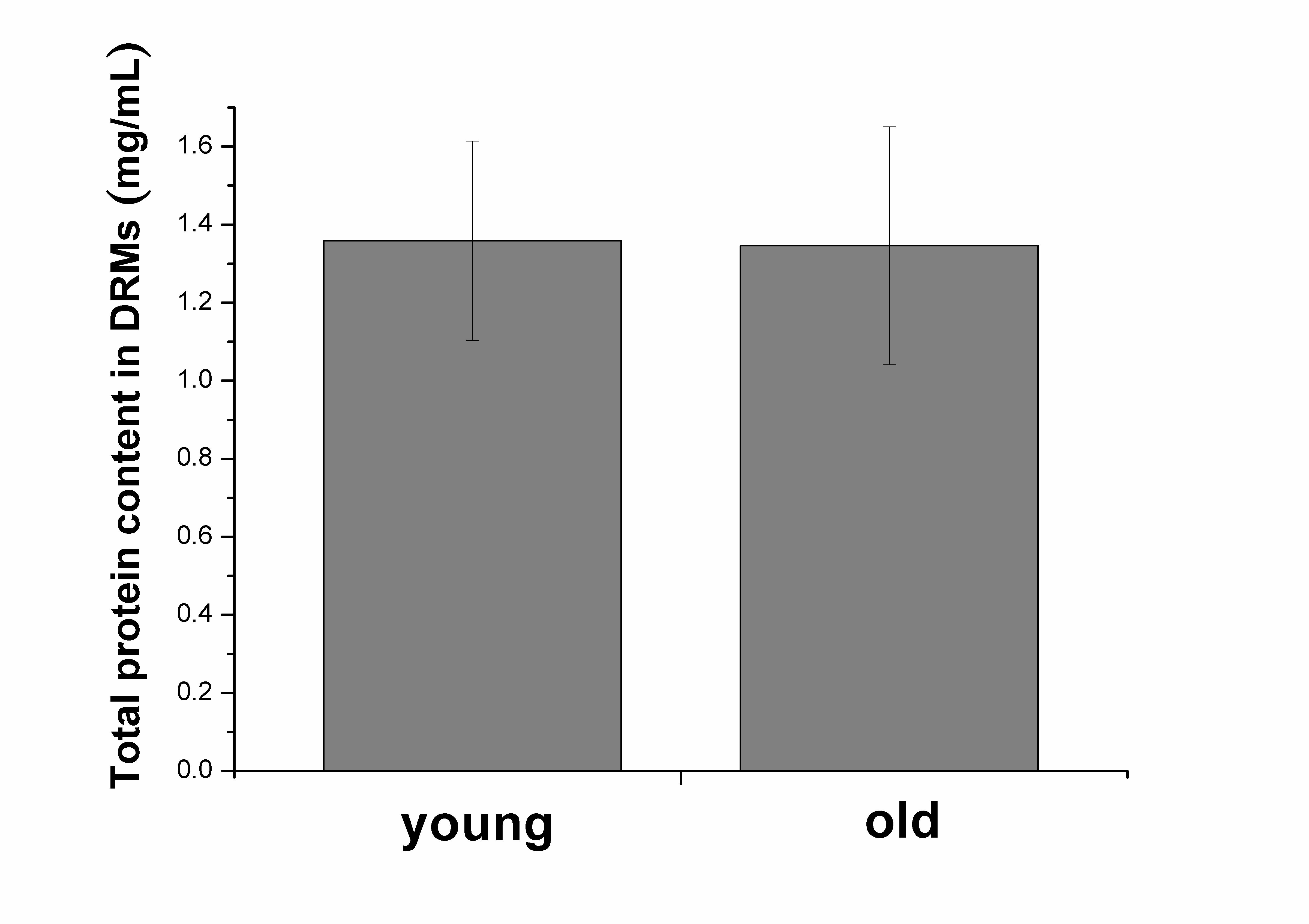

Supplement: Figure S1 — Total protein content in DRMs of hippocampi from young and aging mice. Comparison of total protein levels in DRMs from hippocampi of young adult mice (3-4 months old) with those of aging animals (20-21 months old). (TIF) [file pone.0074244.s001.tif]

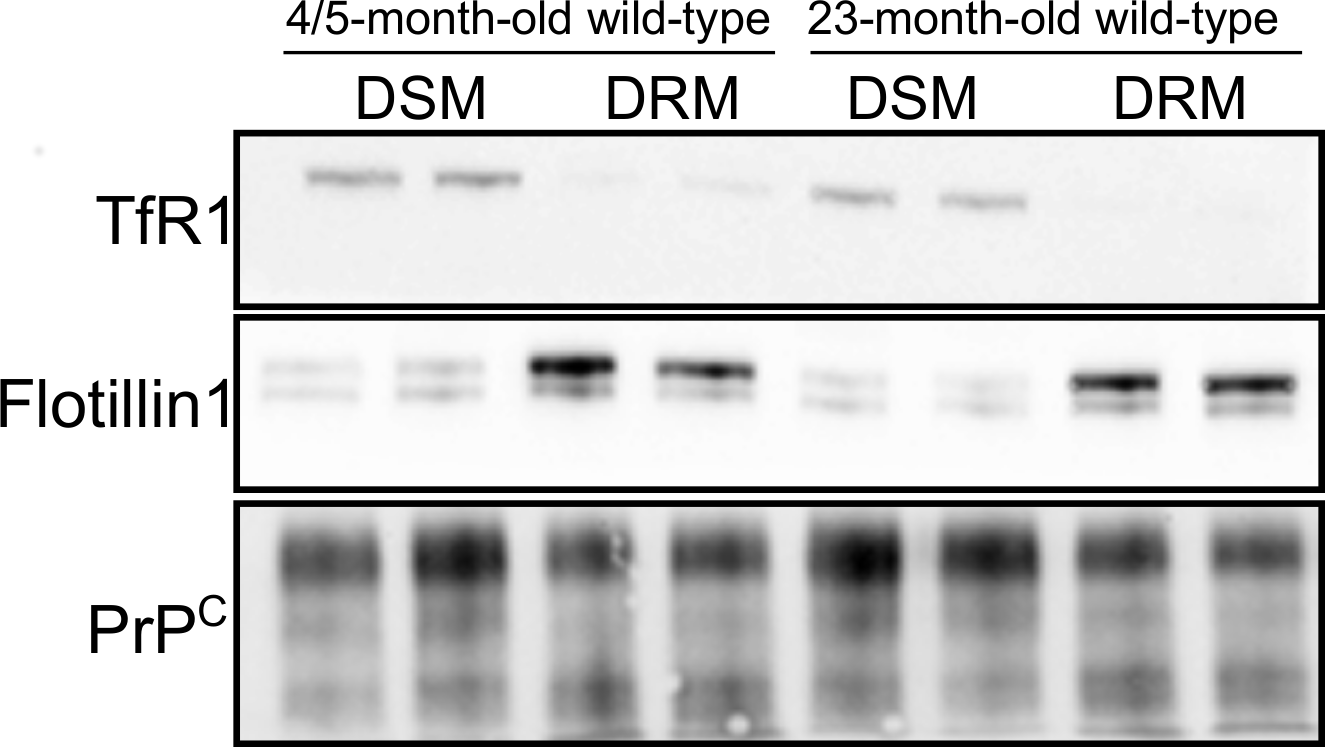

Supplement: Figure S2 — Purity of DRM preparations. Western blot analysis of DRM preparations. Antibodies used: anti-transferrin receptor1 (1:500; Invitrogen, Paisley, UK), mouse monoclonal anti Flotillin1 (1:1,000; BD Transduction), D18, humanized monoclonal anti PrP (1:1,000; InPro Biotechnology, Inc, South San Francisco). (TIF) [file pone.0074244.s002.tif]

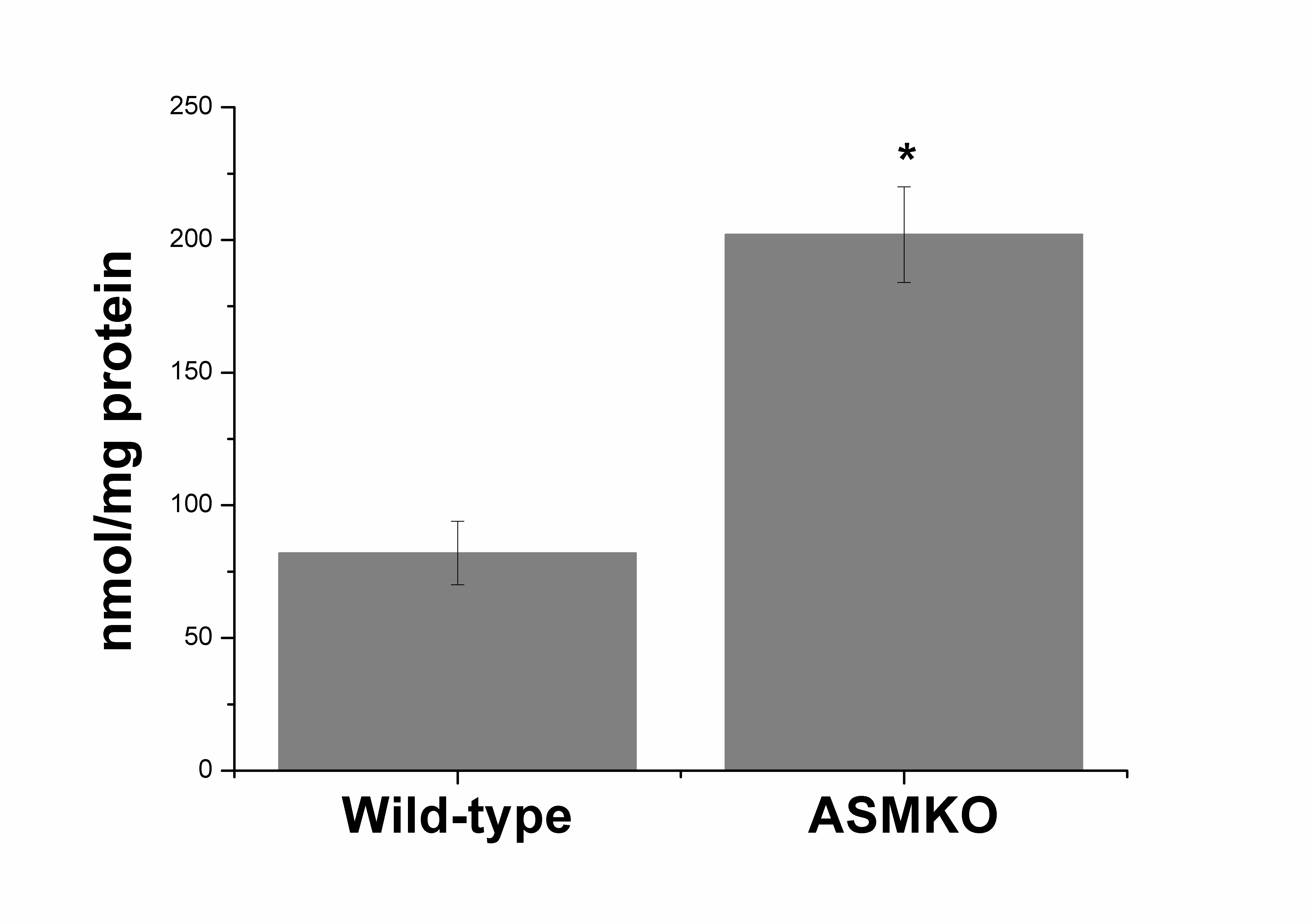

Supplement: Figure S4 — Sphingomyelin in wild-type and ASMKO mice. Comparison of sphingomyelin in 5-month-old wild-type and ASMKO mice. *: p<0.05. (TIF) [file pone.0074244.s004.tif]

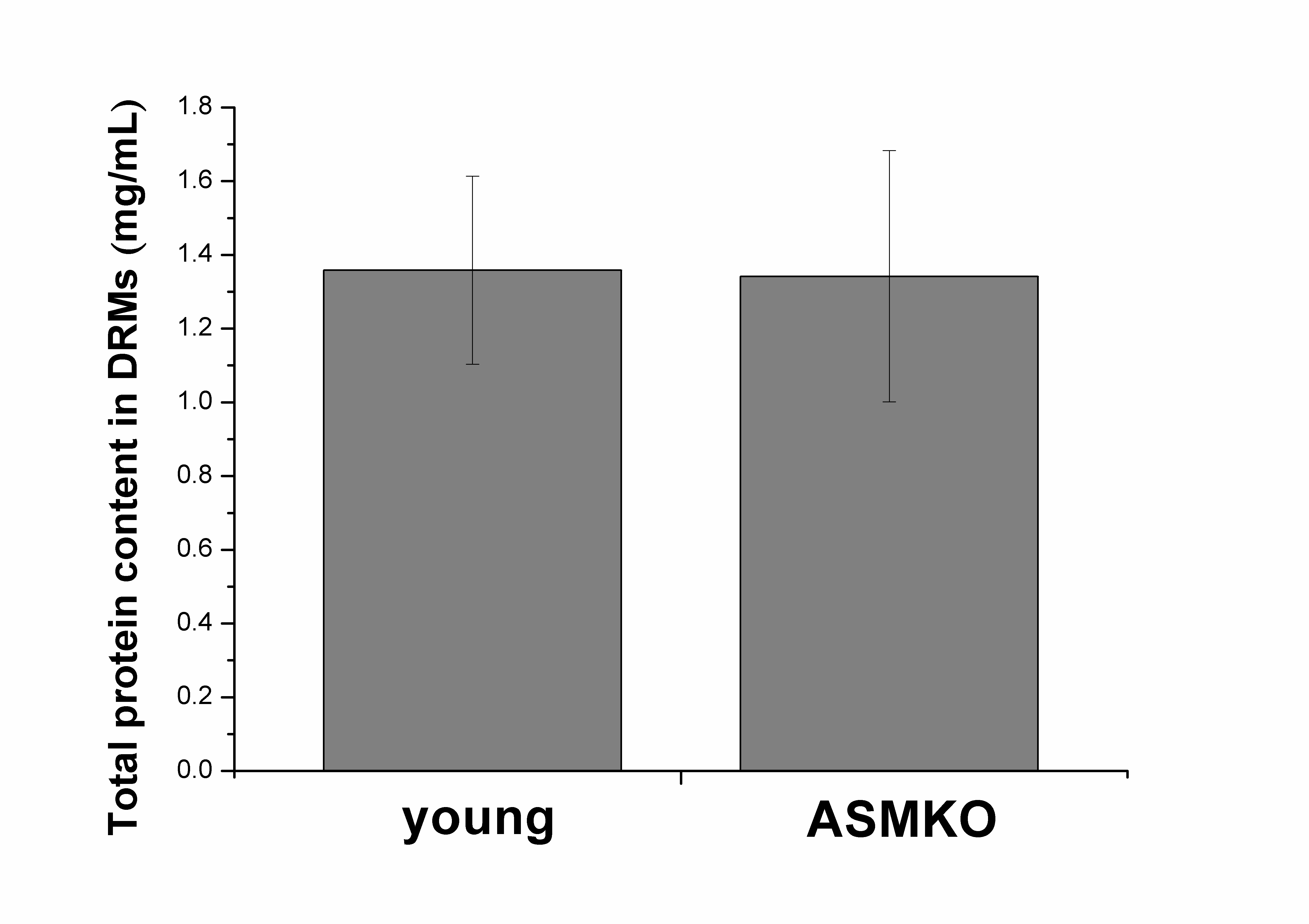

Supplement: Figure S5 — Total protein content in DRMs of hippocampi from young and ASMKO mice. Comparison of total protein levels in DRMs from hippocampi of young adult mice (5 months old) with those of ASMKO littermates. (TIF) [file pone.0074244.s005.tif]

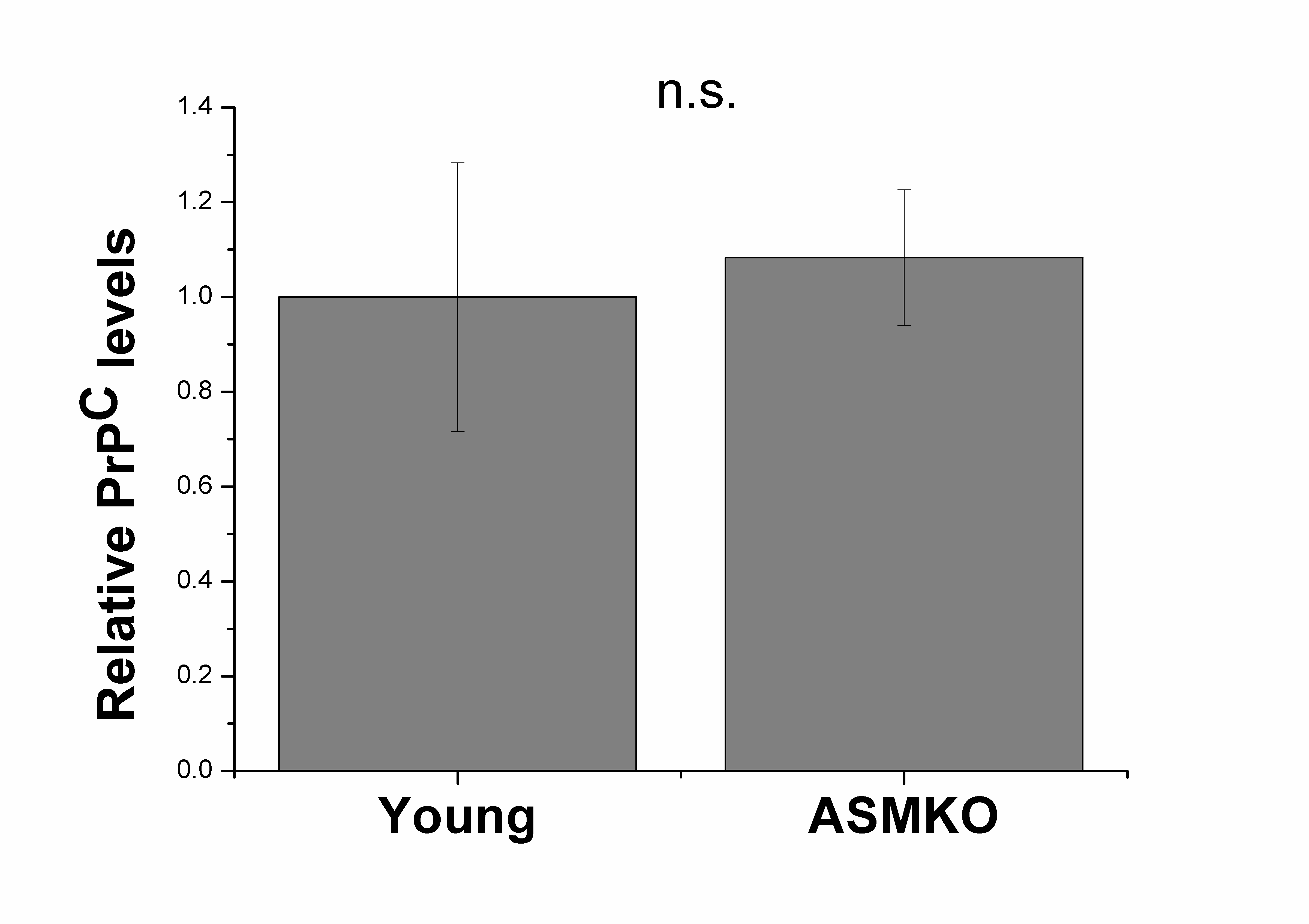

Supplement: Figure S6 — PrPC in DSMs from hippocampal membrane of young wild-type mice compared with age-matched ASMKO mice. Western blot analysis of DSMs prepared from equal amounts of hippocampal extracts from young (4-5 months old) wild-type and ASMKO mice. Antibodies used: D18 (1:1,000; InPro Biotechnology, Inc, South San Francisco). Quantification of relative PrPC amounts from 3 control mice and 3 ASMKO mice. Each data point represents the relative PrPC level normalized over protein loading ± SD. (TIF) [file pone.0074244.s006.tif]

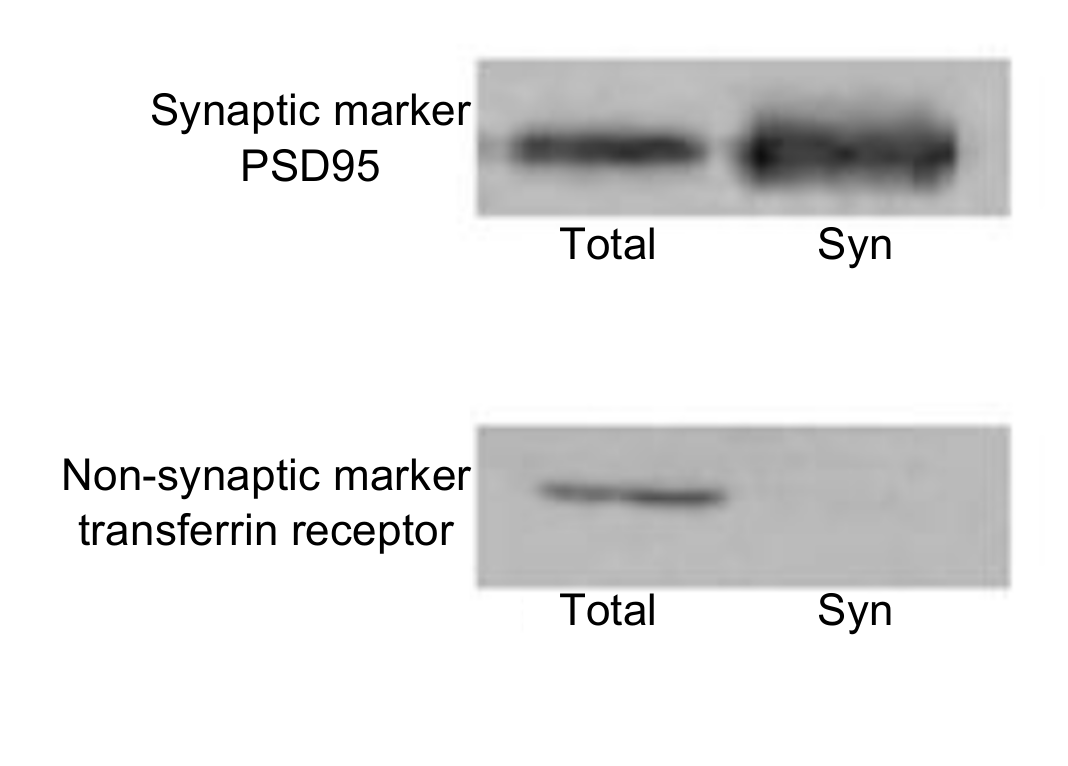

Supplement: Figure S7 — Purity of synaptosomal preparations. Western blot analysis of synaptosomal preparations. Antibodies used: PSD95 (1:100; Sigma), anti-Transferrin receptor (1:500; 13-6800 Invitrogen, Paisley, UK). Total indicates total homogenate, and Syn indicates synaptosomes. (TIF) [file pone.0074244.s007.tif]

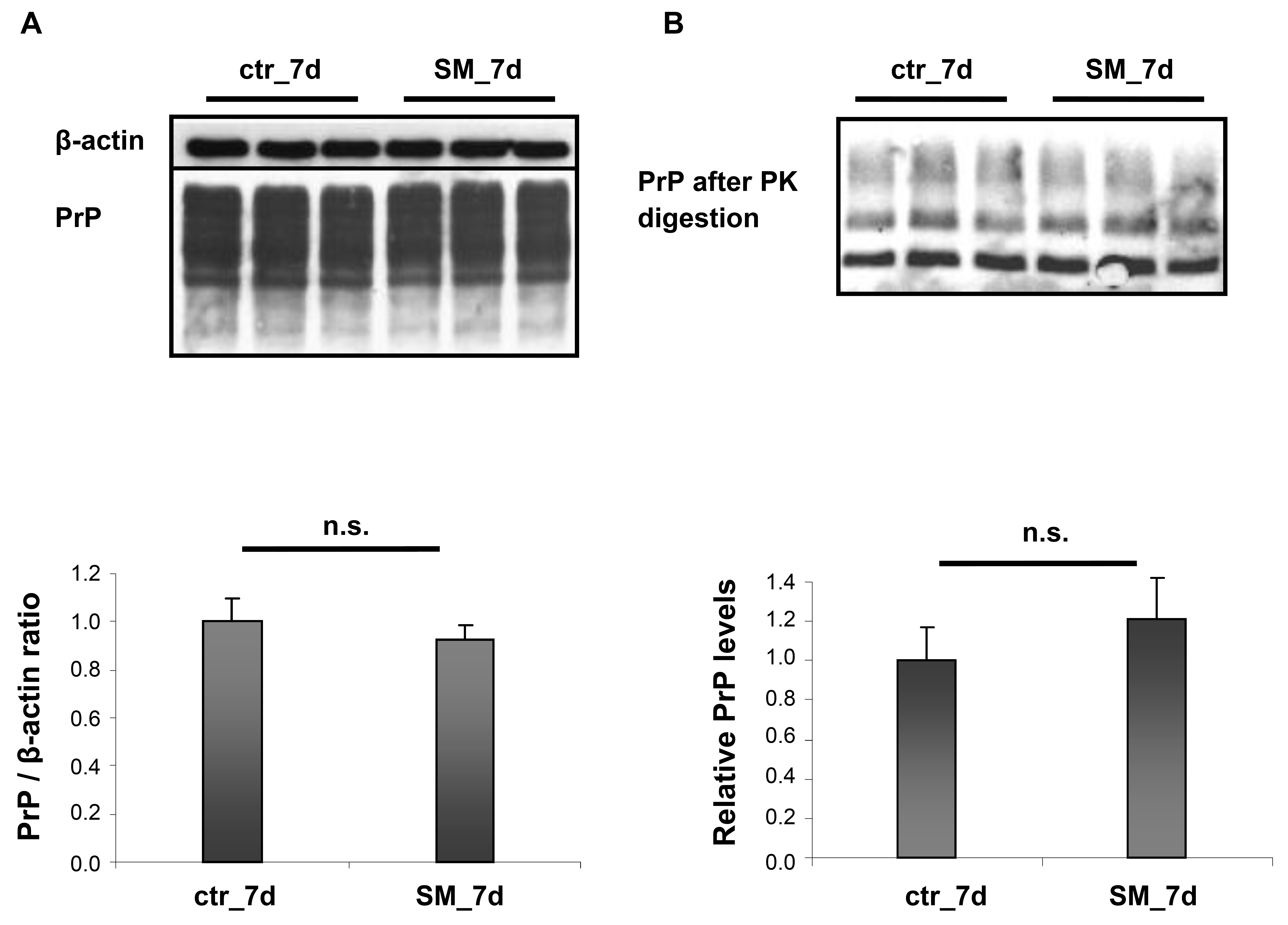

Supplement: Figure S8 — Total PrP and PK-resistant PrP in ScGT1 cells after treatment with SM. ScGT1 cells were treated for 7 days (7d) with sphingomyelin (100 µg/mL). A) Western blot analysis of equal amounts of protein from ScGT1 cells (25 µg per lane). Antibodies used: D18 (1:1,000; InPro Biotechnology, Inc, South San Francisco), mouse monoclonal anti β-actin (1:25,000; Sigma-Aldrich). Each data point represents the mean protein level normalized over β-actin ± SD. B) Western blot analysis of equal amounts of protein from ScGT1 cells (250 µg per lane) after PK digestion. Antibodies used: D18 (1:1,000; InPro Biotechnology, Inc, South San Francisco) and mouse monoclonal anti β-actin (Sigma). Each data point represents the mean protein level normalized over total PrP ± SD. No significant changes in PrP levels were detected in the total protein extracts or in protease-resistant PrP after sphingomyelin treatment. n.s.: not significant. (TIF) [file pone.0074244.s008.tif]

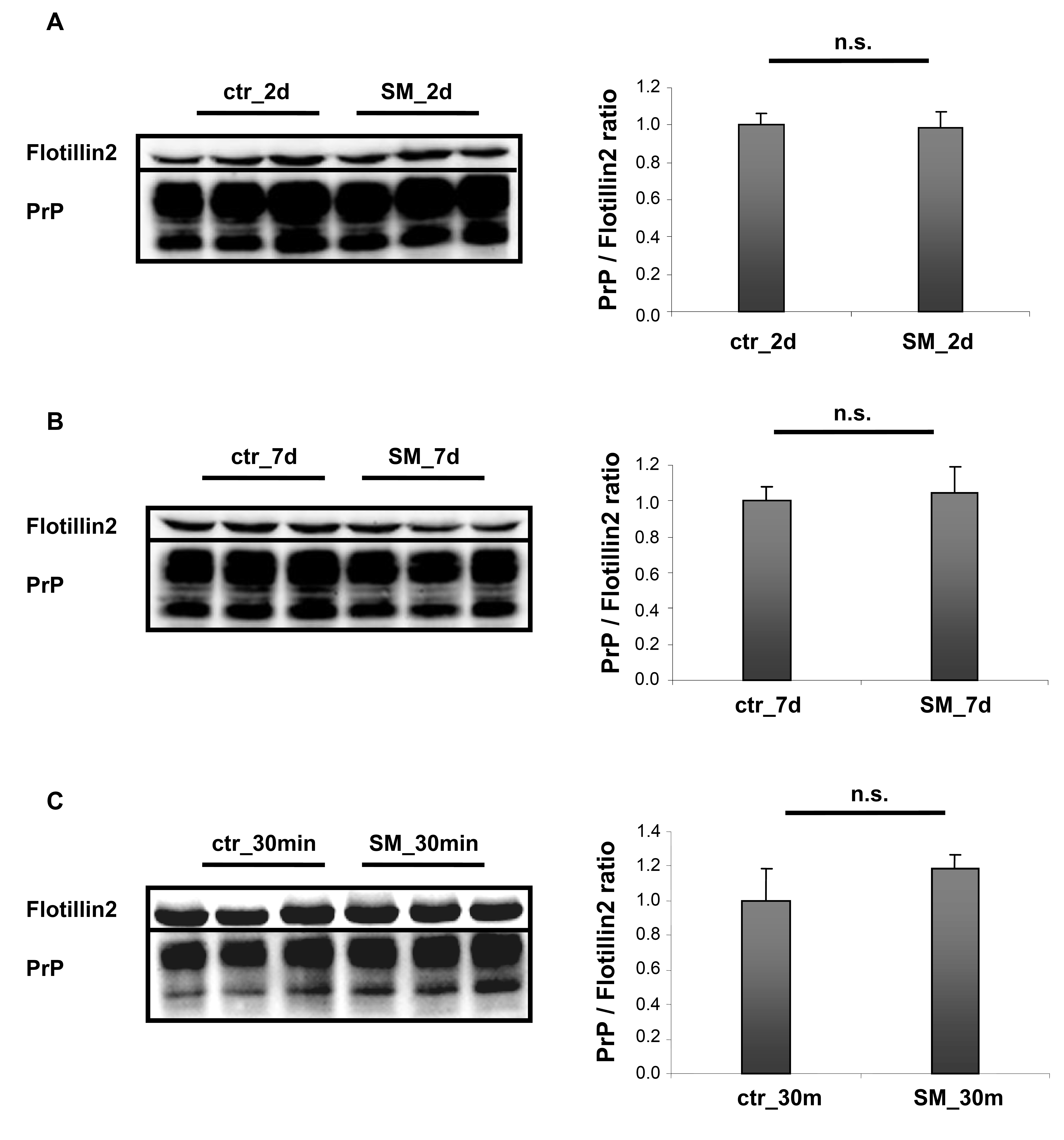

Supplement: Figure S9 — PrPC in DRMs from GT1 cells treated with sphingomyelin. Western blot analysis of DRMs prepared from equal amounts of protein (150 µg of total protein) from GT1 cells treated with 100 µg/ml of sphingomyelin. A) GT1 cells treated for 2 days (2d). B) GT1 cells treated for 7 days (7d). C) GT1 cells treated for 30 minutes (30m). Antibodies used: D18 (1:1,000; InPro Biotechnology, Inc, South San Francisco), mouse monoclonal anti flotillin2 (1:1,000; BD Biosciences). Each data point represents the mean protein level normalized over flotillin2 ± SD. No significant changes in PrP levels could be detected in the DRMs after sphingomyelin long treatment (2d or 7d). After short treatment (30m) PrP showed a tendency to increase, although not significantly. n.s.: not significant. (TIF) [file pone.0074244.s009.tif]
